# Multifaceted Interplay between Hfq and the Small RNA GssA in *Pseudomonas aeruginosa*

**DOI:** 10.1128/mbio.02418-22

**Published:** 2022-12-08

**Authors:** Silvia Santoro, Costanza Paganin, Sara Gilardi, Tarcisio Brignoli, Giovanni Bertoni, Silvia Ferrara

**Affiliations:** a Department of Biosciences, Università degli Studi di Milano, Milan, Italy; University of Rochester

**Keywords:** *Pseudomonas aeruginosa*, sRNAs, Hfq, glucose utilization, pyocyanin, exotoxin A, posttranscriptional regulation

## Abstract

Behind the pathogenic lifestyle of Pseudomonas aeruginosa exists a complex regulatory network of intertwined switches at both the transcriptional and posttranscriptional levels. Major players that mediate translation regulation of several genes involved in host-P. aeruginosa interaction are small RNAs (sRNAs) and the Hfq protein. The canonical role of Hfq in sRNA-driven regulation is to act as a matchmaker between sRNAs and target mRNAs. Besides, the sRNA CrcZ is known to sequester Hfq and abrogate its function of translation repression of target mRNAs. In this study, we describe the novel sRNA GssA in the strain PA14 and its multifaceted interplay with Hfq. We show that GssA is multiresponsive to environmental and physiological signals and acts as an apical repressor of key bacterial functions in the human host such as the production of pyocyanin, utilization of glucose, and secretion of exotoxin A. We suggest that the main role of Hfq is not to directly assist GssA in its regulatory role but to repress GssA expression. In the case of pyocyanin production, we suggest that Hfq interplays with GssA also by converging a positive effect on this pathway. Furthermore, our results indicate that both Hfq and GssA play a positive role in anaerobic growth, possibly by regulating the respiratory chain. On the other hand, we show that GssA can modulate not only Hfq expression at both transcriptional and posttranscriptional levels but also that of CrcZ, thus potentially influencing the pleiotropic role of Hfq.

## INTRODUCTION

The opportunistic pathogen Pseudomonas aeruginosa, responsible for acute and chronic infections, is the leading cause of morbidity and mortality in cystic fibrosis (CF) patients (1). Infections of P. aeruginosa are hard to eradicate due to its extraordinary ability to biofilm formation, remarkable intrinsic resistance to several antibiotics, readiness to acquire resistance through chromosomal mutations, and acquisition of antibiotic resistance genes (2–4). P. aeruginosa lifestyle is a paradigm for adaptation, survival, and persistence (5). Several virulence factors exert crucial roles in P. aeruginosa-induced pathogenesis (5). For example, pulmonary exacerbations of CF patients correlate with upregulation of the expression of genes involved in the production of some destructive virulence factors such as proteases, phenazines, exotoxins, rhamnolipids, and hydrogen cyanide. Among the toxins, exotoxin A is the most potent P. aeruginosa virulence factor (6). A relevant aspect of P. aeruginosa concurring with its pathogenicity is the production of a variety of phenazines (7), including pyocyanin, which is the most abundantly released starting from the early stationary phase. Pyocyanin is a potent diffusible virulence factor causing cell death in infected CF patients via oxidative stress induced by reactive oxygen species. Furthermore, pyocyanin plays a role in facilitating P. aeruginosa biofilm formation (8) by inducing bacterial cell lysis and the release of extracellular DNA (eDNA), intercalating with eDNA, it influences P. aeruginosa cell surface hydrophobicity facilitating bacterial cell-to-cell interaction (aggregation) and ultimately promoting robust biofilm formation (9).

The versatility of the P. aeruginosa that allows it to thrive in multiple environments and hosts has been associated with its relatively large genome (ca. 6 to 7 Mb). At the pangenome level (10), details of which genes are involved in the adaptation to different niches and those implicated in encoding traits of clinical interest have been recently updated (11). However, a large set of genes is not sufficient alone to justify the polyhedral phenotypes of P. aeruginosa. Numerous interlaced transcriptional, posttranscriptional, and posttranslational regulatory mechanisms are indeed in place downstream environmental and host stimuli contributing to the control of the P. aeruginosa versatility and pathogenic potential (12, 13). To add further complexity, there is the emerging notion that intraspecies diversity in the architecture of the regulatory network is important in delineating different behaviors within the three distinct major P. aeruginosa lineages (11), of which the two most populated are represented by reference laboratory strains PAO1 and PA14 (14, 15). This notion was recently strengthened at the posttranscriptional level by Trouillon et al., who demonstrated diversity in core and accessory Hfq interactomes across P. aeruginosa lineages (16). In bacteria, Hfq is one of the main RNA-binding proteins which, being able to target distinct classes of RNAs, can act pleiotropically through several regulatory functions (17–19). The Hfq function that was most studied is its role as an RNA matchmaker, promoting the base-pairing between small RNAs (sRNAs) and their mRNA targets (20). However, Hfq can act as a direct translational repressor of target mRNAs. In P. aeruginosa, this Hfq function occurs very often in concert with the catabolite repression control protein Crc (21), and it was recently shown that there is pervasive targeting of nascent transcripts by Hfq (22). This direct repressive function of Hfq can be antagonized through sequestration by the sRNA CrcZ (23). Therefore, CrcZ acts as a decoy to abrogate Hfq-mediated translational repression, a role implicated in the relief of carbon catabolite repression (23), in enhancing sensitivity toward antibiotics both in PAO1 and in PA14 (24), and in controlling anoxic biofilm formation in PA14 (25). The interplay between Hfq and CrcZ is based on their direct interaction. This raises the question of the magnitude of the decoy potential exerted on Hfq by the vast P. aeruginosa sRNA landscape that was discovered in PAO1 and PA14 (26–28), with an overall number of sRNA hits of 680, and 126 sRNAs in common between the two strains (29). In PAO1, a recent report evidenced a conditional Hfq association with sRNAs, mainly dictated by sRNAs expression levels in the two conditions tested, planktonic versus biofilm growth (30). This suggests an intense sRNA competition for binding to Hfq which modulates its translational repression activity. Besides, there are other indirect aspects (i.e., not due to physical interaction) of the interplay between Hfq and sRNAs that were overlooked and that could be important connectors between transcriptional and posttranscriptional shells of gene regulation. For instance, the influence of Hfq on sRNA stability and/or transcription, and reciprocally the possible modulation of Hfq transcription and translation by sRNAs.

This study focused on a previously not characterized P. aeruginosa sRNA, selected from the panel of sRNAs unique to the PA14 that resulted from our comparative PAO1 versus PA14 survey of sRNAs (27). At the P. aeruginosa pangenome level, this sRNA belongs to the “flexible” genome portion since its gene is present in more than one strain but not all (10). Despite this, it seems structurally wired to the regulation of core processes like glucose utilization, and secretion of virulence factors. For this, we named it GssA, which stands for “Glucose and secretion system-related sRNA A.” Here, we suggest that GssA interplays with Hfq in a polyhedral manner through mechanisms that go beyond physical interaction.

## RESULTS

### A large array of factors concurs in the modulation of the expression of the sRNA GssA.

**FIG 1:**
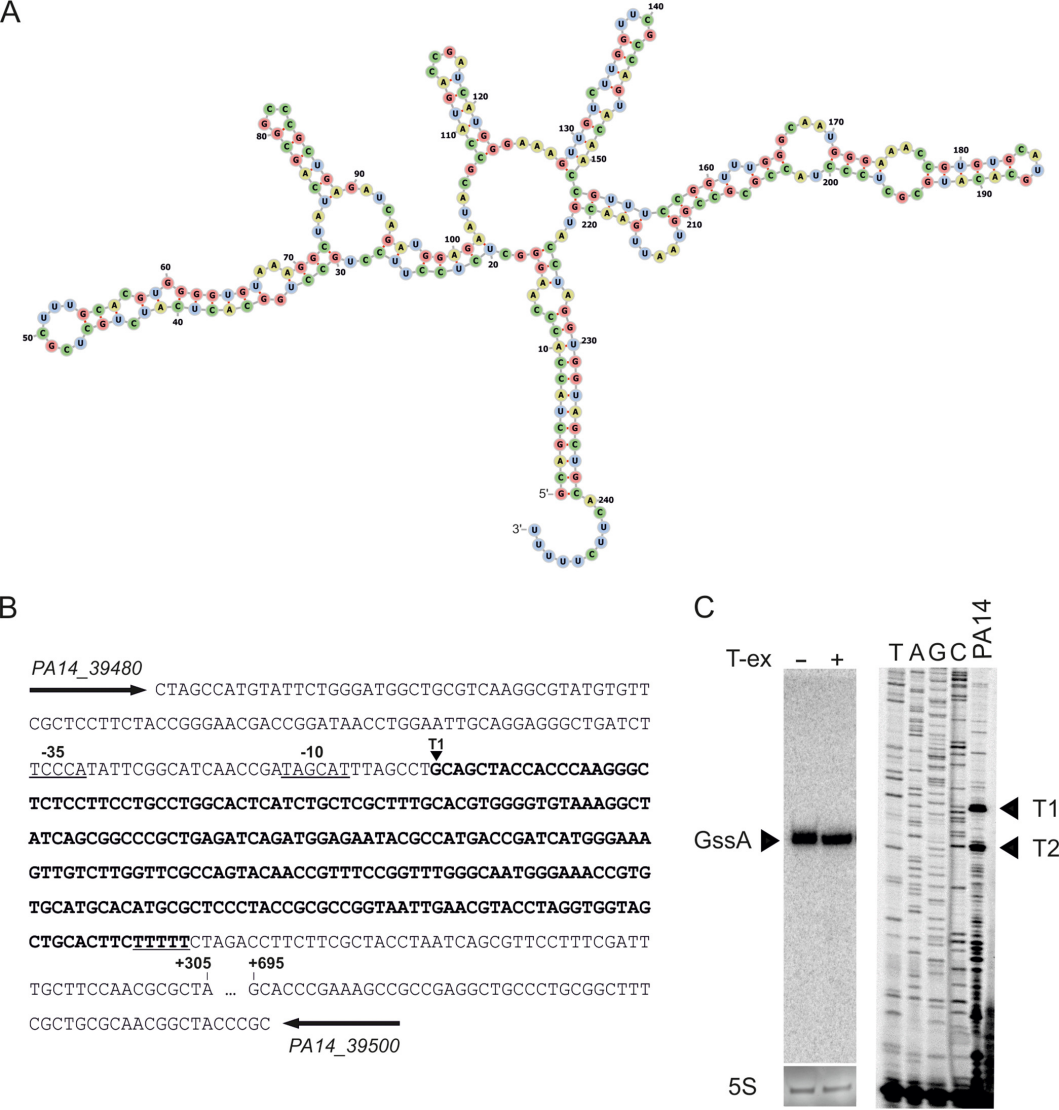
Genomic context of the *gssA* gene and analysis of the 5′ end of GssA RNA. (A) High-confidence prediction of the secondary structure of GssA by the *RNAfold* tool within the Vienna RNA Websuite (56). (B) Sequence of the PA14_39480-to-PA14_39500 intergenic region of PA14 where the *gssA* gene (indicated in boldface) is located. The mapped T1 5′ end of GssA is indicated, while the transcription terminator poly(T) tail is underlined. High-confidence −35 and −10 motifs upstream of T1 are indicated. These motifs were detected by SAPPHIRE (33), a web tool for σ^70^ promoter prediction in Pseudomonas. (C) Northern blot and primer extension analyses of the 5′ end of GssA. Portions (10 μg) of total RNA from PA14 extracted at the end of the exponential growth were untreated (−) or treated (+) with terminator 5′-phosphate-dependent exonuclease (T-ex) and analyzed by Northern blotting. The arrowhead indicates the primary transcript of ~250 nt. The 5′ ends T1 and T2 were mapped by primer extension using 10 μg of total RNA extracted as described above, flanked by a TAGC sequencing ladder.

GssA is a 249-nucelotide (nt) sRNA that is predicted to fold in a high degree of secondary structures (Fig. 1A). It was originally identified as unique to the PA14 in our previous comparative survey of sRNAs in P. aeruginosa PAO1 versus PA14 and formerly called SPA0012 (27). A BLASTN-based search of the *gssA* gene in the https://www.pseudomonas.com/ database (31) allowed to establish perfect gene conservation in 12 P. aeruginosa strains (see Fig. S1). A similar search across all Pseudomonas species returned a high degree of conservation of the *gssA* gene in nine strains of other Pseudomonas species (see Fig. S1). In P. aeruginosa PA14, the *gssA* gene locates in the accessory genome (coordinates 3515399 to 3515700) within the Region of Genomic Plasticity 52 (32) between loci PA14_39480 and PA14_39500 (Fig. 1B). Resistance to treatment with terminator 5′-phosphate-dependent exonuclease (Fig. 1C), which preferentially degrades processed transcripts, indicated that the GssA RNA is a primary transcript. Two 5′ ends, T1 and T2, were detected by primer extension (Fig. 1C). SAPPHIRE (33), a web tool for σ^70^ promoter prediction in Pseudomonas detected high-confidence −35 (TTCCCA) and −10 (TAGCAT) motifs in a proper position from T1 (Fig. 1B). These supposed promoter motifs were perfectly conserved among the P. aeruginosa strains harboring the *gssA* gene (see Fig. S1).

10.1128/mbio.02418-22.2 FIG S1 Conservation of the *gssA* gene and its upstream region. By BLASTN search, the sequence of the PA14 *gssA* gene and its upstream region was queried against the https://www.pseudomonas.com/ database. Eleven P. aeruginosa (PAE) strains were found to harbor a sequence sharing 100% identity with that of PA14. A high degree of identity was found also for nine strains belonging to Pseudomonas mosselii (PMOSS), Pseudomonas chlororaphis subsp. *aurantiaca* (PCHL_SUB), Pseudomonas synxantha (PSYN), Pseudomonas sp FDAARGOS (PspDSFD), Pseudomonas fluorescens (PFLUO), Pseudomonas syringae (PSYR), Pseudomonas mendocina (PMEN), and Pseudomonas fuscovaginae (PFUS). The −35/−10 motifs found by the web tool SAPPHIRE are evidenced in pink while the 5′ end T1 (+1) of GssA determined by primer extension is indicated in boldface. Download FIG S1, JPG file, 1.2 MB. Copyright © 2022 Santoro et al. 2022 Santoro et al. https://creativecommons.org/licenses/by/4.0/ This content is distributed under the terms of the Creative Commons Attribution 4.0 International license.

**FIG 2:**
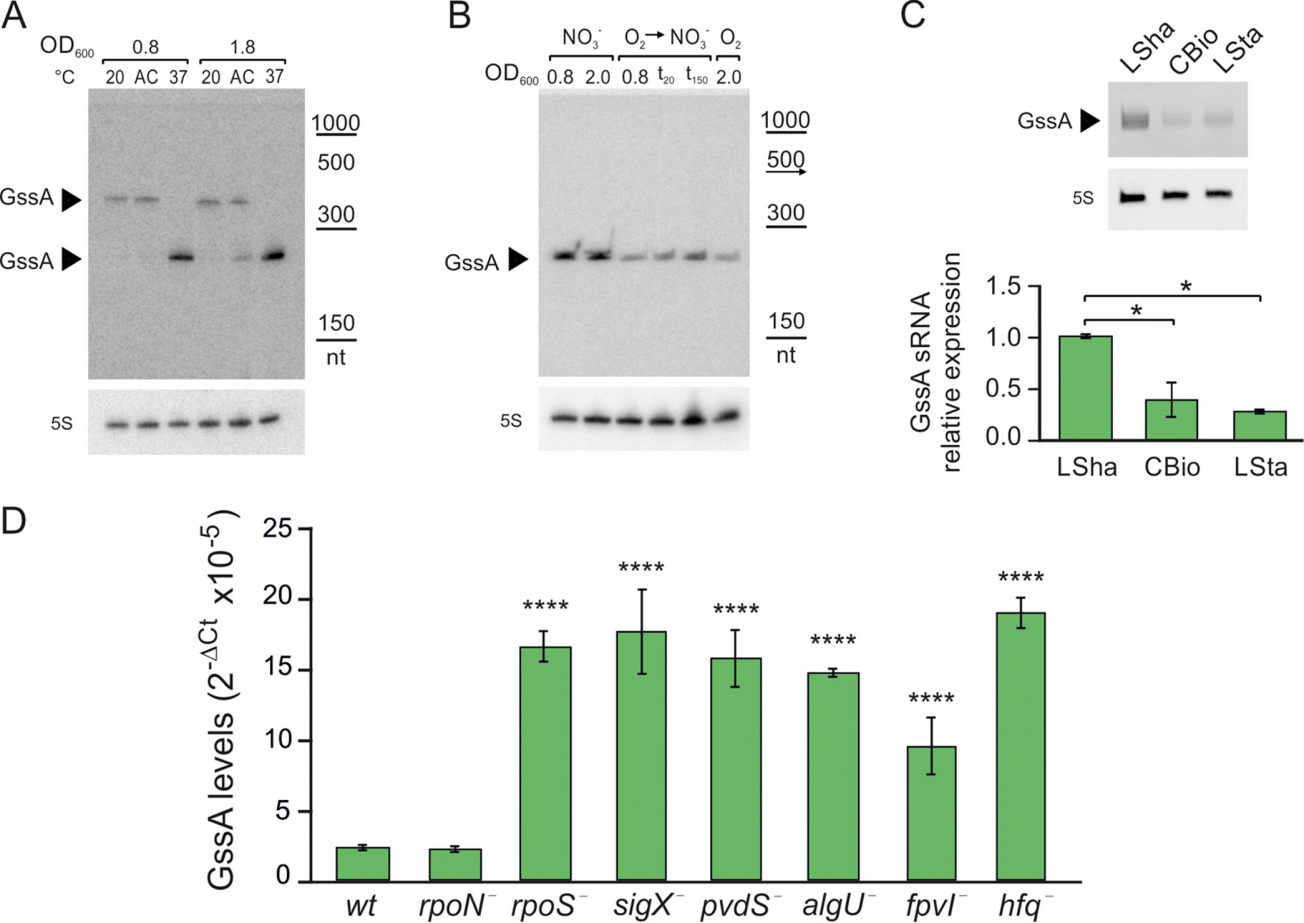
GssA expression is conditioned by temperature, availability of oxygen, planktonic versus aggregative forms of growth, lack of several σ factors, and inactivation of the *hfq* gene. (A) Northern blot analysis of the influence of temperature on GssA expression. PA14 cell samples were taken for total RNA extraction at mid (OD_600_ of 0.8) and late (OD_600_ of 1.8) exponential growth phase in BHI at 20°C, 37°C, or after 20 min of acclimation (AC) from 20 to 37°C. (B) Northern blot analysis of the influence of oxygen availability on GssA expression. PA14 cultures were grown in BHI at 37°C anaerobically (NO_3_^−^), aerobically (O_2_), and aerobically until reaching an OD_600_ of 0.8 and then shifted to anaerobic conditions (O_2_ → NO_3_). Cell samples were taken for total RNA extraction at mid (OD_600_ of 0.8) and late (OD_600_ of 2.0) exponential growth phase and then 20 and 150 min after the shift from aerobic to anaerobic conditions (*t*_20_ and *t*_150_). (C) Effects of the type of growth, either planktonic or aggregative, on GssA abundance analyzed by Northern blotting and quantitative RT-PCR. Cell samples were taken for total RNA extraction from cultures grown at 37°C in liquid LB with shaking overnight (LSha), statically for 48 h (LSta), or on LB-agar in form of colony biofilm (CBio). The calculation by quantitative RT-PCR of the relative expression of GssA in LSha versus CBio and LSta was performed as described by the 2^−ΔΔ^*^CT^* method (57), first normalizing GssA amounts to 16S ribosome RNA (Δ*C_T_*) and then relating the Δ*C_T_* in CBio and LSta to that in LSha (ΔΔ*C_T_*). (D) Effects of inactivating several σ factors and *hfq* genes on GssA abundance. For total RNA extraction and quantitative RT-PCR, cell samples of a panel of previously published σ factor mutants of PA14 and PA14Δ*hfq* were taken from CBio cultures at 37°C on LB-agar. The GssA levels in PA14 and the mutants are displayed graphically as 2^−Δ^*^CT^* values. *, *P* < 0.05; ****, *P* < 0.0001 (calculated by one-way ANOVA with *post hoc* Tukey’s HSD test).

We started the GssA characterization by testing the responsiveness of *gssA* gene expression to temperature shifts from environmental to body temperature and reduced oxygen conditions, signals that P. aeruginosa can sense during infection of CF airways. As shown in Fig. 2A, GssA is expressed as 249-nt RNA at 37°C, at both the midexponential phase (optical density at 600 nm [OD_600_] = 0.8) and the early stationary phase (OD_600_ = 1.8). At 20°C, GssA is expressed at lower levels in a longer version of ~400 nt. Since 249-nt GssA RNA is a primary transcript (Fig. 1C), it is unlikely to derive from the longer version visible at 20°C by processing at the 5′ end. We hypothesized that the σ^70^ promoter mentioned above could be induced by the upward shift in temperature and drive the expression of both the long and the short forms. The latter would result from early termination at 37°C. Alternatively, the long form could originate from an upstream promoter, repressed by an upward temperature shift, and end at the same terminator site as the short version. Besides, at 37°C, the promoter of 249-nt GssA appeared to be responsive to oxygen availability since the GssA levels were higher in anaerobic than aerobic conditions (Fig. 2B). The shift from aerobic to anaerobic conditions indeed induced the GssA expression.

These assessments of GssA responsiveness were performed by extracting RNA from bacterial cells grown under shaking in a flask or stirring in a bioreactor, conditions which are very unlikely to occur in nature during a free lifestyle, and even less during the infection process. Therefore, we aimed to compare GssA levels in planktonic cells grown in liquid medium with shaking (LSha) to those in cells grown in aggregative forms such as colony biofilm (CBio), i.e., growth on the surface of agar medium, which was shown to reproduce most of the biofilm-associated traits (34), and submerged cell aggregates resulting from growth in liquid medium incubated statically (LSta). As shown in Fig. 2C, CBio and LSta elicited comparable GssA levels that were 2.5-fold lower than those at the early exponential phase in LSha. These results suggested that growth conditions such as LSha that disfavor cell aggregation promote GssA expression. This effect is unlikely to be attributable to increased oxygen availability in LSha compared to CBio and LSta since aerobic conditions repress GssA expression (Fig. 2B).

The above *in silico* analysis suggested that *gssA* gene expression is driven by a σ^70^ promoter. However, other alternative σ factors may be involved in its expression. To test this hypothesis, we measured the GssA levels in CBio in a collection of PA14 knockout mutants in the alternative σ factors RpoN, RpoS, SigX, PvdS, FpvI (35), and AlgU (36) with the idea that if the *gssA* gene were under the direct control of one or more of these factors, we would have observed a reduction in its expression. The results of this test were unexpected (Fig. 2D). Except for RpoN, the expression of GssA was much higher in the σ factor mutants than in PA14. This suggested that the *gssA* gene is under the control of one or more transcription factors whose expression depends on the σ factors AlgU, RpoS, SigX, PvdS, and FpvI. To strengthen this hypothesis, we extrapolated from a list of differentially expressed genes in mutants for AlgU, RpoS, SigX, PvdS, and FpvI (35) the transcription factors which resulted in being dysregulated. Furthermore, we listed transcription factors whose promoter region was found to be bound by the same σ factors (35). Using these criteria, we listed several transcription regulators (see Table S1 in the supplemental material) either characterized or presumed. These results suggested that GssA is under the control of various transcriptional factors that, on the whole, respond to several σ factors, allowing a finely modulated expression of GssA in response to different environmental stimuli.

10.1128/mbio.02418-22.8 TABLE S1 Lists of transcriptional regulators under either direct or indirect modulation of a panel of alternative sigma factors. Download Table S1, PDF file, 0.1 MB. Copyright © 2022 Santoro et al. 2022 Santoro et al. https://creativecommons.org/licenses/by/4.0/ This content is distributed under the terms of the Creative Commons Attribution 4.0 International license.

Furthermore, we reasoned whether Hfq could influence the GssA levels as it was found to be abundantly bound to Hfq in PA14 (25). To test a possible effect, we measured the GssA levels in PA14Δ*hfq* (37) in CBio like the analysis of the panel of σ factor mutants. As shown in Fig. 2D, GssA accumulation was strongly increased in PA14Δ*hfq*. This suggested an indirect influence of Hfq on *gssA* gene transcription and/or increased stability of GssA in the lack of Hfq.

Overall, these results indicated that a broad spectrum of conditions impacts the intracellular levels of GssA. Several transcription factors and Hfq are suggested to mediate this wide range of responsiveness. The consequences of the modulation of GssA levels can be reflected in the direct GssA-mediated regulation of individual cellular functions or, indirectly, in the modulation of various genes, as when the direct target is a gene for a regulatory protein, at both the transcriptional and the posttranscriptional level. The results presented below indicate the importance of GssA-mediated regulation in various cellular processes and are compatible with both direct and indirect effects on target genes.

### Deletion of *gssA* derepresses the production of two important *P. aeruginosa* exoproducts and dysregulates several patterns of genes.

**FIG 3:**
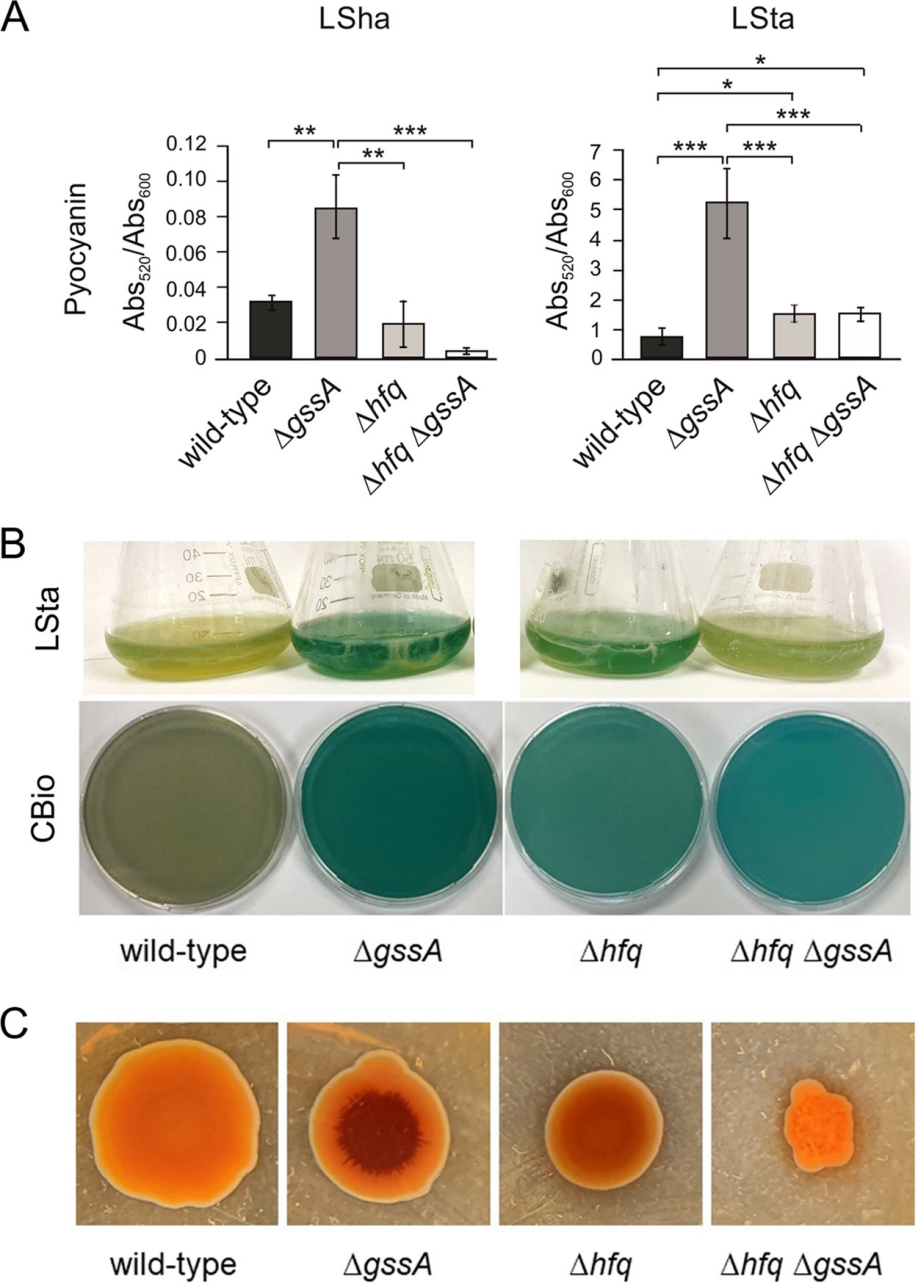
Deletion of GssA derepresses the production of pyocyanin and Pel. (A) Quantification of pyocyanin release in liquid LB by PA14, PA14Δ*gssA*, PA14Δ*hfq*, and PA14Δ*hfq*Δ*gssA* following growth with shaking overnight (LSha) or statically for 48 h (LSta). *, *P* < 0.05; **, *P* < 0.01; ***, *P* < 0.001 (calculated by one-way ANOVA with *post hoc* Tukey’s HSD test). (B) Visual comparison of pyocyanin release by PA14, PA14Δ*gssA*, PA14Δ*hfq*, and PA14Δ*hfq*Δ*gssA* following static growth in liquid LB for 48 h (LSta) or on LB-agar in form of colony biofilm (CBio). (C) Comparison of pigmentation intensity among spots of PA14, PA14Δ*gssA*, PA14Δ*hfq*, and PA14Δ*hfq*Δ*gssA* when grown on the surfaces of Congo red agar plates.

To start the functional characterization of GssA, we deleted the *gssA* gene in PA14 generating the PA14Δ*gssA* mutant. The most surprising phenotype that we observed when we started growing PA14Δ*gssA* was its abundant production of pyocyanin compared to PA14, especially in LSta and CBio. As shown in Fig. 3A, in LSha, pyocyanin released by PA14 was in fact ~50-fold lower than in LSta (note the difference in scale in the two graphs). In LSha, pyocyanin released by PA14Δ*gssA* was 2.5-fold higher than PA14. In LSta, pyocyanin levels released by PA14Δ*gssA* were ~5-fold higher than PA14, although quantifiable only visually, the same difference was also evident in CBio (Fig. 3B). Hence, GssA appeared to play a repressive role in pyocyanin production under all conditions tested, although with a greater magnitude in LSta and CBio than in LSha. This role can be carried out through direct GssA-mediated regulation of genes involved in the biosynthesis of pyocyanin or their regulators. As shown in Fig. S2, the higher pyocyanin-releasing phenotype in PA14Δ*gssA* than PA14 could be reverted, i.e., complemented, by the expression of GssA from the plasmid vector pGM931 (36). This ruled out possible indirect effects of the deletion of the *gssA* gene that we generated.

10.1128/mbio.02418-22.3 FIG S2 GssA expressed from a plasmid vector can complement the Δ*gssA* mutation-induced phenotype of increased pyocyanin production. Pyocyanin release by PA14 and PA14Δ*gssA* harboring pGM931, and PA14Δ*gssA* harboring pGM*gssA* was quantified in liquid LB following static growth for 48 h. *, *P* < 0.05; ***, *P* < 0.001 (calculated by one-way ANOVA with *post hoc* Tukey’s HSD test). Download FIG S2, JPG file, 0.1 MB. Copyright © 2022 Santoro et al. 2022 Santoro et al. https://creativecommons.org/licenses/by/4.0/ This content is distributed under the terms of the Creative Commons Attribution 4.0 International license.

Given the strong upregulation effect of the *hfq* deletion on GssA levels (Fig. 2D), the release of pyocyanin in PA14Δ*hfq* could be expected to be inhibited by the higher GssA levels. On the other hand, if the repressive function of GssA requires Hfq (i.e., as RNA chaperone), pyocyanin production in PA14Δ*hfq* could be expected to be derepressed as in PA14Δ*gssA*. As shown in Fig. 3A and B, the pyocyanin release by PA14Δ*hfq* in LSta and CBio was moderately derepressed if compared to PA14Δ*gssA*, consistent with a combination of upregulation of GssA and lack of Hfq-mediated RNA chaperone activity in PA14Δ*hfq*. If this were the case, we expected upregulation of pyocyanin release similar to PA14Δ*gssA* in the double mutant PA14Δ*hfq*Δ*gssA*. However, in PA14Δ*hfq*Δ*gssA*, the release of pyocyanin did not equal that of PA14Δ*gssA* and was comparable to that of PA14Δ*hfq*. This evidence suggested that the lower pyocyanin release levels in PA14Δ*hfq* compared to PA14Δ*gssA* were not due to a combination of elevated GssA levels and the absence of Hfq chaperone activity, but rather to the lack of a positive Hfq regulatory role. In LSha (Fig. 3A), the pattern of pyocyanin release by PA14Δ*hfq* and PA14Δ*hfq*Δ*gssA* compared to PA14 and PA14Δ*gssA* resembled that in LSta and CBio. These results together suggest that independently from growth conditions pyocyanin production undergoes negative control of GssA. However, Hfq can have a positive role in itself, directly or indirectly, on the pathway of pyocyanin production.

Another interesting phenotype of PA14Δ*gssA* was the overproduction of Pel. Since PA14 is incapable of Psl production (38), Pel is the primary biofilm matrix exopolysaccharide in this strain. As shown in Fig. 3C, when cultivated on agar plates with Congo red, a dye known to bind Pel (39), PA14Δ*gssA* spots turned dark red in the central portion compared to the PA14, indicating again, like pyocyanin production, a negative role of GssA. The pattern of Pel production in PA14Δ*hfq* and PA14Δ*hfq*Δ*gssA* was similar to that of pyocyanin, suggesting also in this case an interplay between GssA and Hfq.

To obtain a preliminary picture at the transcriptional level of the set of genes linked to the regulatory activity of GssA, we analyzed the global transcription profile of PA14Δ*gssA* strain in comparison with PA14 by an transcriptome sequencing (RNA-seq) approach, extracting total RNA at OD_600_ of 2, corresponding to early stationary phase, when the two strains were grown in brain heart infusion (BHI) rich medium, a condition in which, at the time of its identification (27), a relevant expression of GssA was noted. With the experimental design and statistical stringency indicated in Text S1, this analysis indicated 241 differentially expressed genes (DEGs), which included 140 genes with a functional classification (see Table S2A), and 101 locus tags annotated as hypothetical proteins or sharing similarities with characterized proteins (see Table S2B). Interestingly, several functional patterns of genes were dysregulated in PA14Δ*gssA* (see Table S2A). Among the upregulated genes in PA14Δ*gssA*, the most populated pattern included 21 genes, both structural and regulatory, involved in glucose transport and metabolism (see Fig. S3). Moreover, we found 10 genes linked to the type VI secretion system (T6SS), mainly belonging to the HCP secretion island I, 7 genes involved in the respiratory chain, including 5 *nir* genes and 1 *nor* gene for denitrification, 10 genes linked to fatty acid biosynthesis and metabolism, and 5 genes for pyochelin biosynthesis. Among the downregulated genes, the most populated pattern consisted of 16 genes of the respiratory chain, including (i) the whole set (13 genes) of *nuo* genes for NADH dehydrogenase (NADH:quinone oxidoreductase) NDH-1, which is largely redundant with the other NADH dehydrogenase NDH-2 under aerobic conditions but, conversely, NDH-1 is required for robust growth under anaerobic conditions since compensatory upregulation of NDH-2 does not occur in NDH-1 deletion strains (40), (ii) the *snr1*, for cytochrome *c* Snr1, and (iii) the *cioAB* gene for the cyanide-insensitive terminal oxidase. Populated patterns of downregulated genes were also 7 genes linked to the type III secretion system (T3SS), including *exoT* and *exoY* genes for ExoT and ExoY exotoxins, respectively, 5 genes for trehalose metabolism, 6 genes for glycogen transport and metabolism, 8 genes for type IVb pilus assembly, including 5 *tad* genes, *rcpC*, and *flp*, and 3 genes for the Chaperon-Usher Pathway CupE pilus assembly, and 7 genes for amino acid metabolism.

10.1128/mbio.02418-22.1 TEXT S1 Detailed materials and methods describing bacterial strains and culture conditions, plasmid construction and mutant generation, RNA isolation and analysis, RNA sequencing and data analysis, sRNA-mRNA interaction *in vivo*, pyocyanin and exotoxin A quantification, and the Congo red binding assay. Download Text S1, PDF file, 0.2 MB. Copyright © 2022 Santoro et al. 2022 Santoro et al. https://creativecommons.org/licenses/by/4.0/ This content is distributed under the terms of the Creative Commons Attribution 4.0 International license.

10.1128/mbio.02418-22.4 FIG S3 Organization and regulation of genes involved in glucose uptake and metabolism in P. aeruginosa. The DEGs in the PA14 Δ*gssA* mutant are represented in gray. The other genes are represented in white. Genes encoding the repressors, also upregulated in PA14Δ*gssA*, are represented in red. The red lines indicate negative regulations. Download FIG S3, TIF file, 1.7 MB. Copyright © 2022 Santoro et al. 2022 Santoro et al. https://creativecommons.org/licenses/by/4.0/ This content is distributed under the terms of the Creative Commons Attribution 4.0 International license.

10.1128/mbio.02418-22.9 TABLE S2 Transcription profile of PA14Δ*gssA* strain in comparison with PA14 by an RNA-seq approach. (A) Differentially expressed genes in PA14Δ*gssA* compared to PA14. (B) Differential expressed genes in PA14Δ*gssA* strain compared to PA14 annotated as coding hypothetical proteins or sharing similarity with characterized proteins. Download Table S2, PDF file, 0.4 MB. Copyright © 2022 Santoro et al. 2022 Santoro et al. https://creativecommons.org/licenses/by/4.0/ This content is distributed under the terms of the Creative Commons Attribution 4.0 International license.

### GssA and Hfq interplay at the level of glucose metabolism and anaerobic respiration.

**FIG 4:**
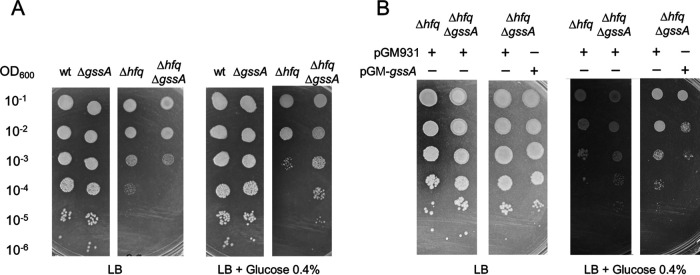
Deletion of *hfq* gene impairs glucose utilization and the simultaneous lack of GssA rescues this defect. (A) Portions (2 μL) of cultures of PA14, PA14Δ*gssA*, PA14Δ*hfq*, and PA14Δ*hfq*Δ*gssA*, serially diluted 10-fold, were spotted onto agar plates with LB and LB-glucose media and incubated at 37°C for 48 h. (B) Portions (2 μL) of cultures of PA14Δ*hfq* and PA14Δ*hfq*Δ*gssA* harboring the control vector pGM931, and PA14Δ*hfq*Δ*gssA* harboring pGM-*gssA*, serially diluted 10-fold, were spotted onto agar plates with LB and LB-glucose media and incubated at 37°C for 72 h.

The results above suggested a role of GssA in repressing the transport and metabolism of glucose. Glucose is considered a nonpreferred carbon source eliciting intermediate carbon catabolite repression (CCR) in the hierarchical management of carbon sources by P. aeruginosa (41, 42), with succinate and malate at the top of the preferred carbon sources and exerting elevated CCR. To assess whether *gssA* deletion could influence glucose utilization, we compared the growth of PA14 and PA14Δ*gssA* in M9-glucose or M9 to other carbon and energy sources, as well as LB or LB-glucose. As shown in Fig. 4A and Fig. S4, PA14Δ*gssA* grows like PA14 under all conditions. In contrast, the lack of Hfq in PA14Δ*hfq* specifically affected growth in the presence of glucose. This impairment was alleviated in PA14Δ*hfq*Δ*gssA*, suggesting that the growth defect in the presence of glucose of PA14Δ*hfq* could be due to increased expression of GssA in a Δ*hfq* background, as shown in Fig. 2D, thus causing the repression of glucose utilization. As further evidence, the overexpression of GssA from the plasmid pGM931 in PA14Δ*hfq*Δ*gssA* represses the growth efficiency in the presence of glucose, making it comparable to that observed in PA14Δ*hfq* (Fig. 4B).

10.1128/mbio.02418-22.5 FIG S4 Efficiency of growth of PA14, PA14Δ*gssA*, PA14Δ*hfq*, and PA14Δ*hfq*Δ*gssA* on M9 medium supplemented with different carbon sources, as indicated. Portions (2 μL) of cultures serially diluted 10-fold were spotted and incubated at 37°C for 48 h. M9-glucose medium recapitulates the same effect of LB-glucose, showing that the deletion of *hfq* gene impairs glucose utilization and the simultaneous lack of GssA rescues this defect. Download FIG S4, TIF file, 0.4 MB. Copyright © 2022 Santoro et al. 2022 Santoro et al. https://creativecommons.org/licenses/by/4.0/ This content is distributed under the terms of the Creative Commons Attribution 4.0 International license.

**FIG 5:**
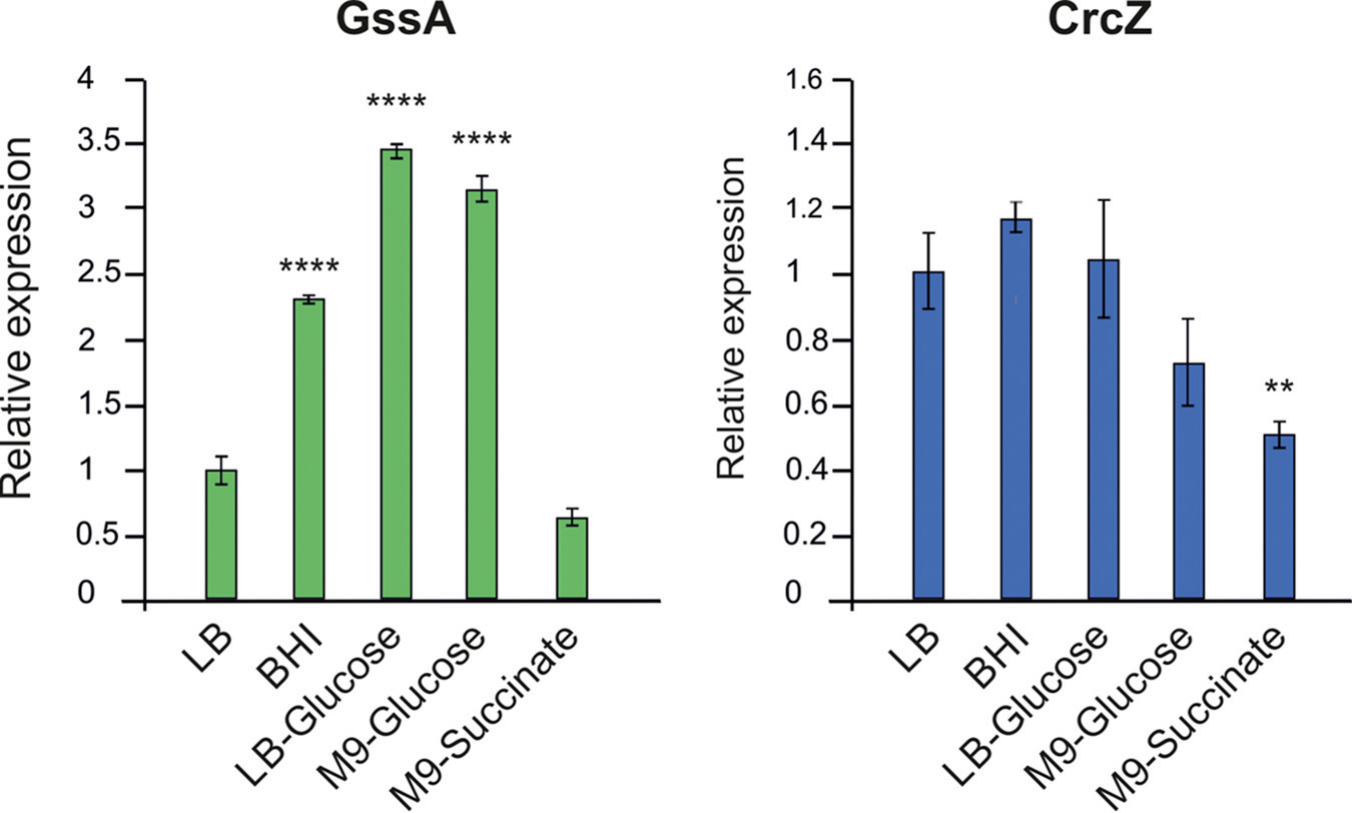
GssA expression is stimulated in glucose-containing media. PA14 cultures were grown overnight at 37°C in liquid with shaking in the indicated media, and then cell samples were taken for total RNA extraction. The expression of GssA and CrcZ in the different media relative to LB was calculated by quantitative RT-PCR using the 2^−ΔΔ^*^CT^* method. **, *P* < 0.01; ****, *P* < 0.0001 (calculated by one-way ANOVA with *post hoc* Tukey’s HSD test). Note that the GssA responsiveness to glucose in CBio and LSta is shown in Fig. S5.

The possible responsiveness of GssA to the presence of glucose could be added to the effect observed in PA14Δ*hfq*. Therefore, GssA levels were evaluated in PA14 grown in LSha in LB, LB-glucose, BHI (which contains 0.2% glucose), M9-glucose, and M9-succinate. As shown in Fig. 5, GssA expression is induced by the presence of glucose, spanning from 2.5-fold in BHI to ~3.5-fold in LB-glucose and M9-glucose. On the contrary, in M9-succinate the GssA levels were lower than in the presence of glucose and similar to those observed in LB. As a control, the levels of the sRNA CrcZ, which is one of the master regulators involved in CCR in P. aeruginosa (12, 42), were evaluated under the same conditions. As expected, CrcZ levels were moderately or not influenced by glucose, whereas succinate repressed its expression (Fig. 5). Responsiveness to glucose was not influenced by the growth conditions since increased GssA levels compared to LB were also observed in BHI in both CBio and LSta (see Fig. S5). These results indicate that GssA is glucose -induced and, at the same time, participates in the downregulation of glucose utilization. GssA may play a regulatory role in one branch of an incoherent feed-forward loop that regulates glucose utilization.

10.1128/mbio.02418-22.6 FIG S5 GssA expression is stimulated in the glucose-containing medium BHI independently from the type of growth. In either LB or BHI, PA14 cultures were grown overnight in liquid with shaking (LSha), overnight on the surface of medium-agar (CBio), or for 48 h in liquid statically (LSta), and then cell samples were taken for total RNA extraction. By quantitative RT-PCR, the expression of GssA in BHI relative to LB was calculated by using the 2^−ΔΔ^*^CT^* method. ****, *P* < 0.0001 (calculated by one-way ANOVA with the *post hoc* Tukey’s HSD test). Download FIG S5, JPG file, 0.1 MB. Copyright © 2022 Santoro et al. 2022 Santoro et al. https://creativecommons.org/licenses/by/4.0/ This content is distributed under the terms of the Creative Commons Attribution 4.0 International license.

**FIG 6:**
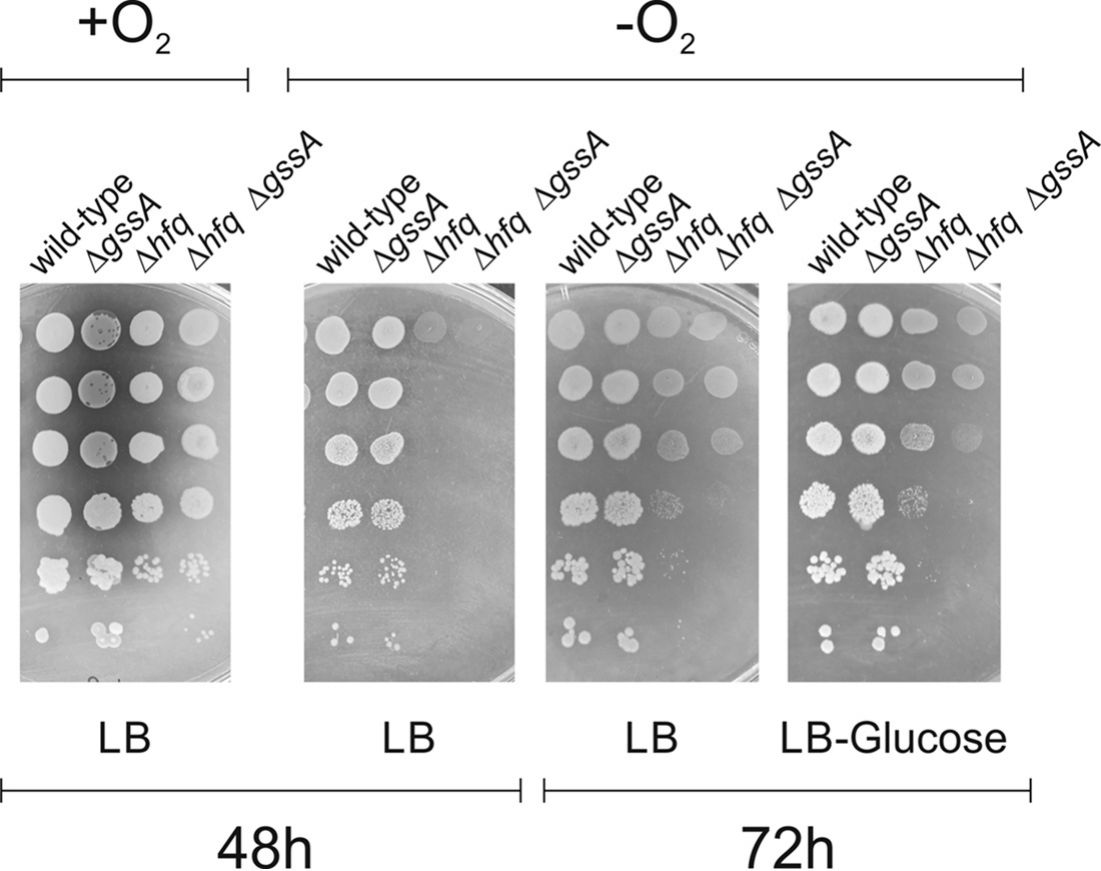
Hfq and GssA have a convergent positive role in anaerobic growth. Portions (2 μL) of cultures of PA14, PA14Δ*gssA*, PA14Δ*hfq*, and PA14Δ*hfq*Δ*gssA*, serially diluted 10-fold, were spotted onto LB or LB-glucose agar plates supplemented with 100 mM KNO_3_ to allow anaerobic respiration. Plates were incubated under aerobic (+O_2_) or anaerobic (−O_2_) conditions at 37°C for 48 to 72 h.

The downregulation of *nuo* genes in PA14Δ*gssA* (see Table S2A), which also occurs in PA14Δ*hfq* in anoxic biofilm (25), suggested a role of GssA in anaerobic respiration and an interplay with Hfq also in this context. To test this, the growth of PA14, PA14Δ*gssA*, PA14Δ*hfq*, and PA14Δ*hfq*Δ*gssA* was assessed under anaerobic conditions when spotted on agar plates and observed after 48 h of incubation. As shown in Fig. 6, PA14Δ*gssA* grows in anaerobic conditions like PA14. As expected, PA14Δ*hfq* growth was impaired in anaerobiosis (25). As evidenced by a longer incubation of 78 h, PA14Δ*hfq*Δ*gssA* was even more compromised than PA14Δ*hfq* (Fig. 6), indicating a positive joint role of GssA and Hfq on the expression of *nuo* genes, with, however, a predominant role of Hfq in this regulation. No influence on this regulation by the presence of glucose was evident.

### GssA can influence the expression of CrcZ and that of Hfq at both transcription and posttranscription levels.

**FIG 7:**
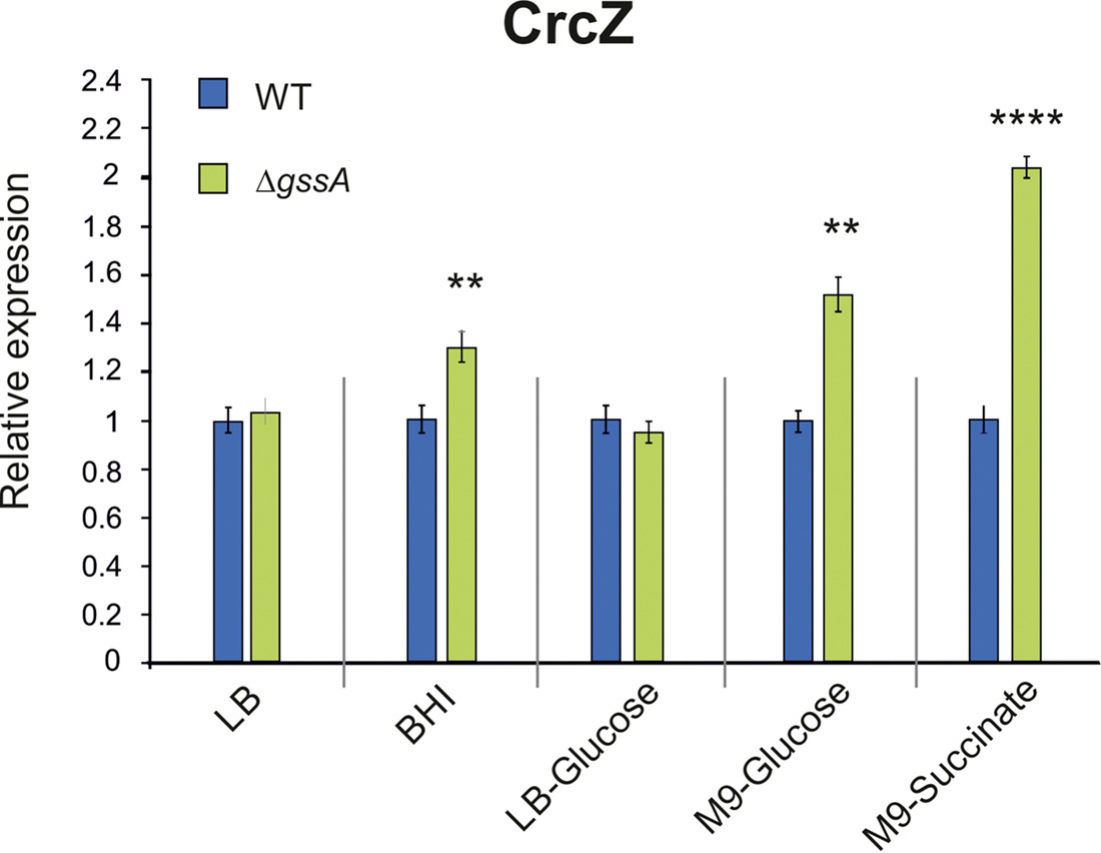
CrcZ levels are affected by the deletion of GssA in a growth medium-dependent manner. PA14 and PA14Δ*gssA* cultures were grown overnight at 37°C in liquid with shaking in the indicated media, and cell samples were taken for total RNA extraction. By quantitative RT-PCR, the expression of CrcZ in the different media relative to LB was calculated by using the 2^−ΔΔ^*^CT^* method. **, *P* < 0.01; ****, *P* < 0.0001 (calculated by one-way ANOVA with *post hoc* Tukey’s HSD test).

**FIG 8:**
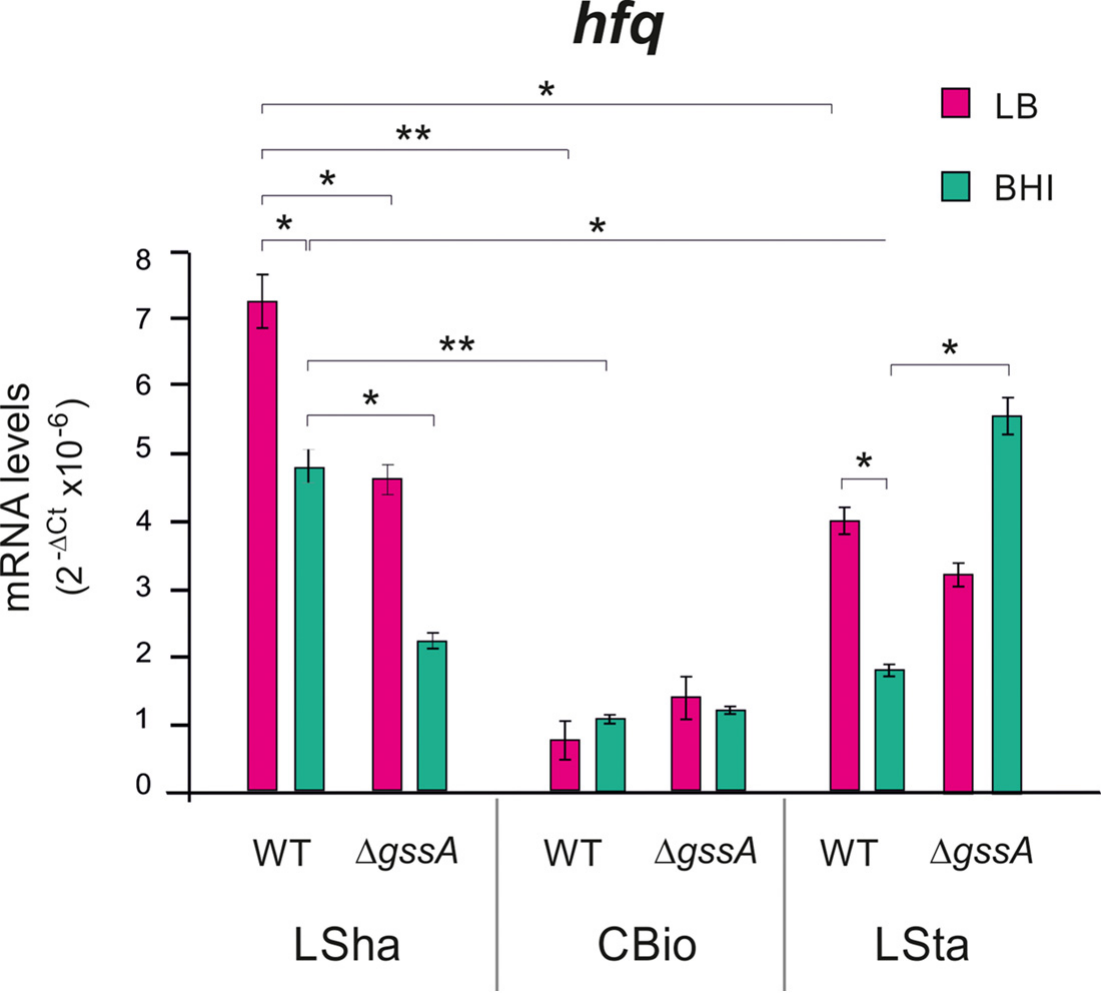
*hfq* mRNA levels are affected by the deletion of GssA in a growth condition-dependent manner. In LB or BHI at 37°C, PA14 and PA14Δ*gssA* cultures were grown overnight in liquid with shaking (LSha), overnight on the surface of medium-agar (CBio), or for 48 h in liquid statically (LSta), and then cell samples were taken for total RNA extraction. By quantitative RT-PCR, the levels of *hfq* mRNA in PA14 and PA14Δ*gssA* are displayed graphically as 2^−Δ^*^CT^* values. *, *P* < 0.05; **, *P* < 0.01 (calculated by one-way ANOVA with *post hoc* Tukey’s HSD test).

The Hfq/GssA interplay in glucose utilization could base on the negative regulation of Hfq on GssA. We wondered whether a reciprocal GssA-mediated regulation of Hfq could also occur through CrcZ, which exerts a decoy effect on Hfq (12, 42) and/or with regulations at both transcription and posttranscription levels of *hfq* gene. First, the CrcZ levels in PA14 were compared to those in PA14Δ*gssA* in different growth media in LSha. As shown in Fig. 7, in BHI, M9-glucose, and M9-succinate the CrcZ levels were upregulated in PA14Δ*gssA* by 30, 50, and 100%, respectively. This indicated that GssA can negatively influence the expression of CrcZ, with a stronger effect under CCR, i.e., in the presence of succinate. Second, the levels of *hfq* mRNA in PA14 and PA14Δ*gssA* were compared in LSha, CBio, and LSta, both in LB and in BHI. As shown in Fig. 8, CBio strongly repressed *hfq* mRNA levels, and no influence of either the Δ*gssA* mutation or the medium could be appreciated. In contrast, in LSha the *hfq* mRNA levels were overall higher and downregulated in PA14Δ*gssA* in both LB and BHI. Independently of GssA, the effect of BHI was to repress *hfq* mRNA levels. In LSta, the *hfq* mRNA levels were generally intermediate between LSha and CBio, and the repression by BHI was still present in PA14. No effect of Δ*gssA* mutation was observed in LB. On the contrary, in BHI the *hfq* mRNA levels were 3-fold upregulated in PA14Δ*gssA*. These results indicated that GssA can influence Hfq expression at the transcription level in a growth condition-dependent manner. In LSha, the GssA-mediated influence is positive independently of the medium used. In LSta, however, the GssA-mediated influence is negative and is evident only in BHI.

**FIG 9:**
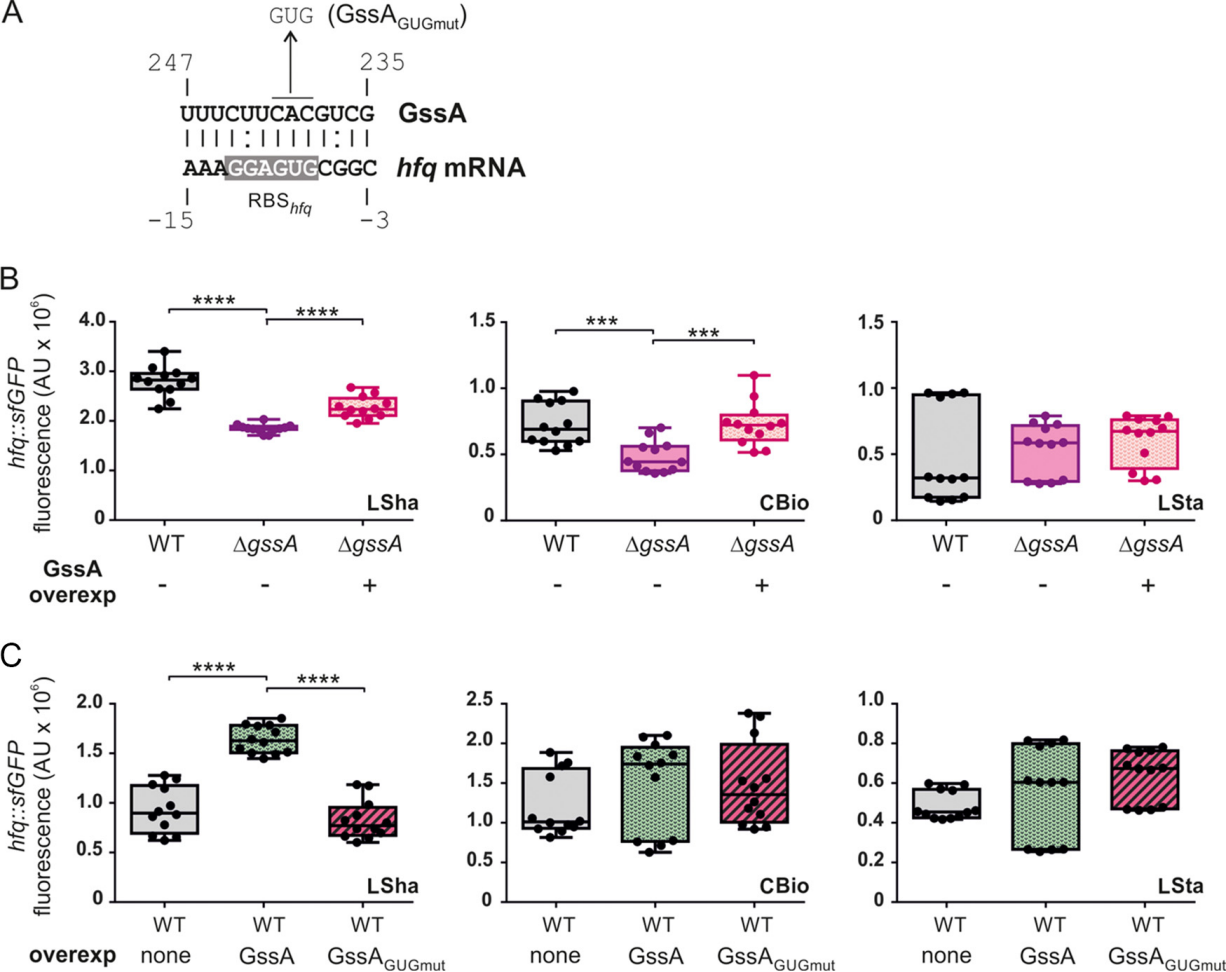
GssA can enhance *hfq* mRNA translation in a growth-dependent manner. (A) Prediction by IntaRNA software (43) of the base-pairing interactions between GssA and *hfq* mRNA. Sequence coordinates are the same as in Fig. 1A for GssA and refer to the +1 translation start site for *hfq* mRNA. The base triplet change in GssA for generating the variant GssA_GUGmut_ is indicated, and the ribosome binding site (RBS_hfq_) of *hfq* mRNA is highlighted. (B) Comparison of the fluorescence expressed by the translational fusion *hfq*::*sfGFP* in PA14 and PA14Δ*gssA* strains harboring pGM931 (–) and PA14Δ*gssA* harboring pGM-*gssA* (+). (C) Comparison of fluorescence resulting from the translational fusion *hfq*::*sfGFP* in PA14 combined with the control vector pGM931 and the plasmids pGM-*gssA* or pGM-*gssA_GUGmut_*. The data are expressed in arbitrary units (AU) as the mean (*n* = 12) of the ratio FI_485_/_535_/Abs_595_ ± the SD. ***, *P* < 0.001; ****, *P* < 0.0001 (calculated by one-way ANOVA with *post hoc* Tukey’s HSD test).

To make an even more complete analysis, which included the posttranscriptional level, we used the IntaRNA software (43) to predict interactions between GssA and *hfq* mRNA and found a potential annealing site, including the ribosome-binding site (RBS) of *hfq* mRNA (Fig. 9A). We then generated a translational fusion between the 5′ UTR of *hfq* mRNA and sfGFP and tested it in PA14Δ*gssA* versus PA14 in LSha, CBio, and LSta. As shown in Fig. 9B, *gssA* deletion caused a decrease in sfGFP activity in LSha and CBio that could be restored by GssA overexpression from the pGM931 plasmid vector. These results suggested a positive direct regulatory role of GssA on *hfq* mRNA translation. To further evaluate this, we overexpressed GssA in PA14 and observed an increase in sfGFP activity in LSha (Fig. 9C) that was consistent with a positive role. Furthermore, overexpression of the variant GssA_GUGmut_, for which the predicted GssA/*hfq* mRNA interaction is expected to be destabilized, did not elicit any increase in GFP activity in LSha. In CBio, no significant increase in sfGFP activity was observed following the GssA overexpression. Since GssA levels are lower in CBio than in LSha (Fig. 2C), we hypothesize that the extra expression from pGM391 in CBio may not be sufficient to exceed the levels of sfGFP observed in the absence of overexpression, as was the case in LSha.

Taken together, these results suggest that GssA may participate in the modulation of Hfq-mediated global regulation both through a negative role on CrcZ levels and by influencing Hfq expression at both transcriptional and translational levels.

### GssA can influence the levels of *toxA* mRNA and interplay with Hfq for exotoxin A secretion.

**FIG 10:**
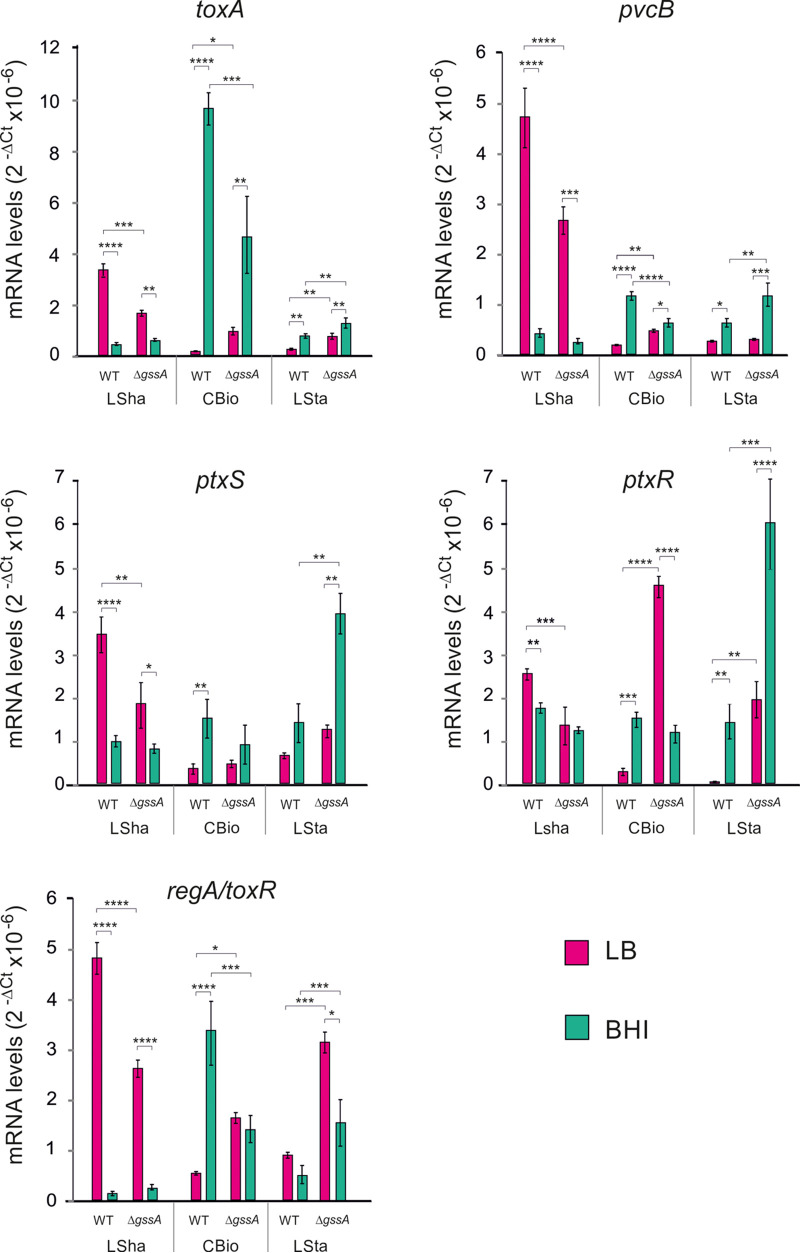
GssA can influence the levels of *toxA* mRNA and those of *pvcB*, *ptxS*, *ptxR*, and *regA* and *toxR.* In LB or BHI at 37°C, PA14 and PA14Δ*gssA* cultures were grown overnight in liquid with shaking (LSha), overnight on the surface of medium-agar (CBio), or for 48 h in liquid statically (LSta), and then cell samples were taken for total RNA extraction. The levels of mRNAs for the different genes in PA14 and PA14Δ*gssA* are displayed graphically as 2^−Δ^*^CT^* values determined by quantitative RT-PCR. *, *P* < 0.05; **, *P* < 0.01; ***, *P* < 0.001; ****, *P* < 0.0001 (calculated by one-way ANOVA with *post hoc* Tukey’s HSD test).

The regulation of the expression of glucose utilization genes and that of *toxA* encoding exotoxin A are linked (44, 45). Since GssA is glucose-responsive and influences several glucose utilization genes negatively (see Table S2A), we wondered whether GssA could play a role, at the transcription level, in the regulation of *toxA*. We added as further readout in this transcription analysis the *pvcB* gene of the *pvcABCD* operon which is involved in the synthesis of the pyoverdine chromophore and subject to regulation similar to *toxA* (46). These analyses were conducted in LSha, CBio, and LSta, both in LB and BHI, comparing PA14 and PA14Δ*gssA*. As shown in Fig. 10, the overall patterns of expression of *toxA* and *pvcB* mRNAs were consistent. Taking PA14 in LB as a reference and comparing the planktonic (LSha) versus the aggregative forms (CBio, and LSta) of growth, the latter repressed the mRNA levels of the two genes. Furthermore, an opposite growth type-dependent glucose regulation was observed in which, in BHI, mRNAs were repressed under LSha, while they were induced in CBio and LSta. This effect was strong for *toxA* in CBio, with an ~24-fold increase in BHI versus LB. In PA14Δ*gssA*, the loss of GssA influenced the levels of *toxA* and *pvcB* mRNAs in a condition-dependent manner. In LSha, the mRNA reduction of ~2-fold in PA14Δ*gssA* compared to PA14 in LB suggested a GssA positive role. No indication of the role of GssA in BHI was obtained possibly due to the strong repression. In CBio, the mRNA increase of ~6-fold in LB suggests a negative GssA role, while in BHI it can be observed the opposite. In LSta, the levels of the two mRNAs were higher in PA14Δ*gssA* compared to PA14 in both LB and BHI, indicating in this case a coherent negative role in both media. To get a wider picture, we analyzed under the same conditions the mRNA levels of transcription factors implicated in the regulation of genes for the use of glucose and *toxA*, such as PtxS, PtxR (45) and RegA/ToxR (47) (Fig. 9). In PA14, aggregate growth is a condition that lowers mRNA levels also for these three genes. Growth in BHI represses them in LSha, while they are induced in CBio and LSta, with the only exception of *regA*/*toxR* mRNA in LSta. Comparing PA14 and PA14Δ*gssA* in LSta, a large overlap in the pattern of the levels of the mRNAs could be noted between the mRNA levels of these three regulators and those of *toxA* and *pvcB*, i.e., an increase in PA14Δ*gssA* in both LB and BHI, thus suggesting a negative role of GssA independent of the presence of glucose. Also in the case of LSha, there was an extended overlap of responses to the lack of GssA, which consisted in a decrease of mRNA levels in LB, suggesting a positive GssA role. As above, there were no evident effects in BHI, possibly due to strong repression from other factors under this condition. In CBio, there was a match of the effects of the Δ*gssA* mutation on the *regA*/*toxR* mRNA levels compared to *toxA* and *pvcB* mRNAs. Under this condition, no apparent effect of GssA on *ptxR* mRNAs in BHI and on *ptxS* mRNAs in both LB and BHI.

**FIG 11:**
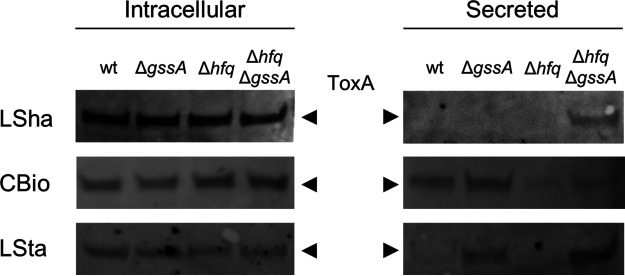
GssA and Hfq interplay in the regulation of secretion of exotoxin A. In LB at 37°C, PA14, PA14Δ*gssA*, PA14Δ*hfq*, and PA14Δ*hfq*Δ*gssA* cultures were grown overnight in liquid with shaking (LSha), overnight on the surface of medium-agar (CBio), or for 48 h in liquid statically (LSta). For LSha and LSta, cells were directly separated from the growth medium by centrifugation. For CBio, samples of cells were collected from the agar surface by inoculation loops, resuspended in PBS, and then pelleted by centrifugation. Cell lysates and supernatants (the growth media for LSha and LSta and PBS for CBio) were analyzed by Western blotting with anti-exotoxin A antibodies for detecting intracellular and extracellular levels of exotoxin A (ToxA), respectively.

With this complex transcriptional scenario in the background, we then aimed to evaluate the possible influence of GssA on the intracellular production and secretion of exotoxin A. We have undertaken this analysis in LB to limit the number of surrounding parameters and observe only the effects due to the type of growth and the presence or absence of GssA, aware of the fact, however, that in LB there was a transcriptional modulation of *toxA* by GssA (Fig. 10). Furthermore, we aimed to evaluate an interplay with Hfq using the PA14Δ*hfq* and PA14Δ*hfq*Δ*gssA* strains. As shown in Fig. 11, intracellular levels of exotoxin A in PA14 were not affected by either Δ*gssA* and Δ*hfq* single mutations or the Δ*hfq* Δ*gssA* double mutation. Thus, protein levels appeared not to mirror GssA-dependent regulation of *toxA* mRNA (Fig. 10), although consistency in protein levels and mRNA levels can be observed when comparing growth conditions, i.e., they were higher in LSha and lower in CBio and LSta. The scenario was completely different when we analyzed the secreted protein (Fig. 11). In PA14, PA14Δ*gssA*, and PA14Δ*hfq* it was not detectable in LSha, while it was in PA14Δ*hfq*Δ*gssA*. In contrast, in CBio and LSta, exotoxin A was detectable in PA14, and its abundance followed an opposite trend in the two single mutants, being increased in PA14Δ*gssA* and decreased in PA14Δ*hfq*. Strikingly, in PA14Δ*hfq*Δ*gssA* the levels of the protein were restored to those of the single Δ*gssA* mutant. Taken together, these results indicate that GssA participates in the downregulation of exotoxin A secretion. The interplay with Hfq can be the same as proposed for glucose utilization, i.e., the role of Hfq would be to repress the expression of GssA.

## DISCUSSION

**FIG 12:**
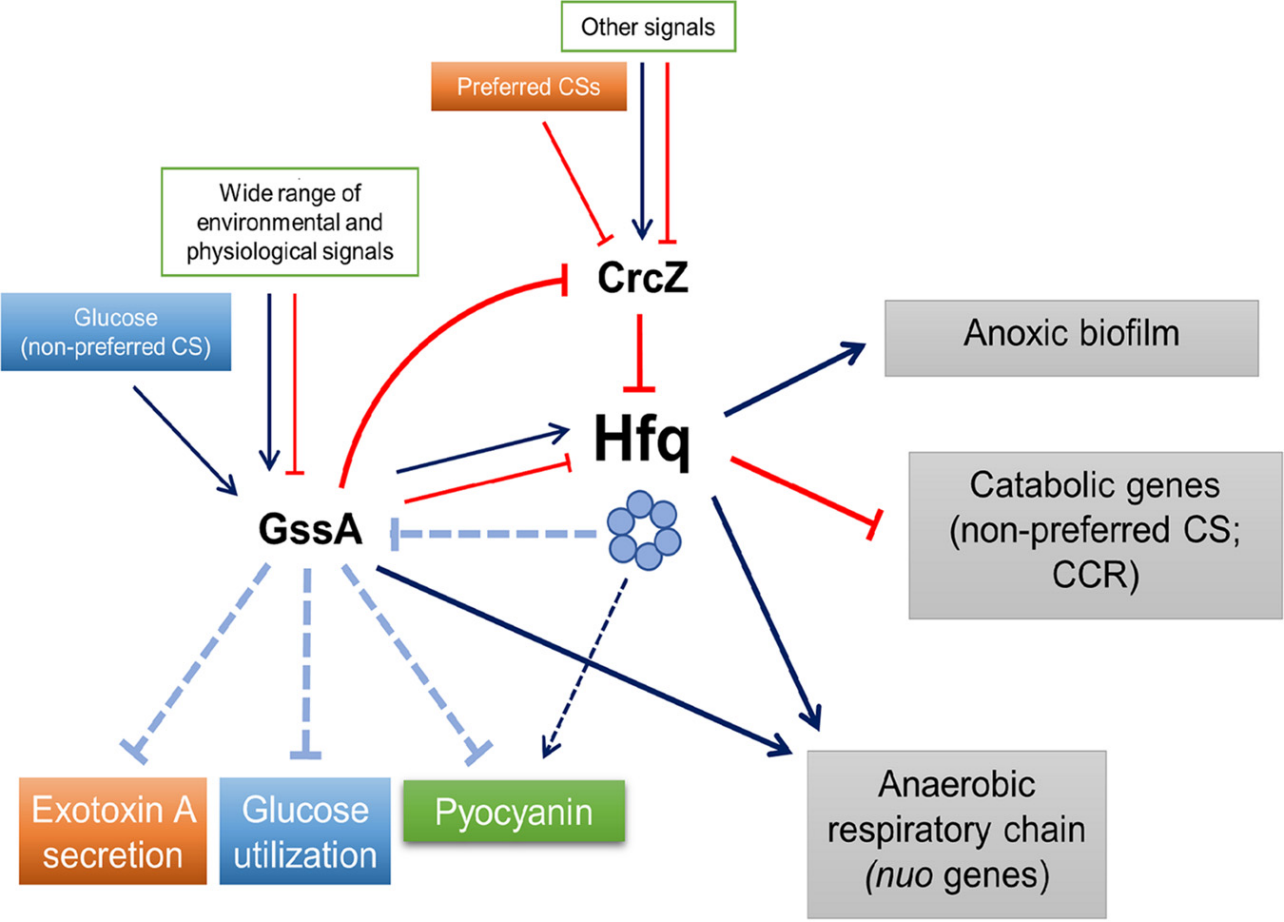
Schematic representation of the interplay between GssA and Hfq. Hfq is seen in the middle as a central regulatory hub. One main effect of Hfq is to repress GssA and this can influence cellular functions related to the interaction with the host such as the secretion of exotoxin A and the utilization of glucose under the effect of stimuli that influence the regulatory activity of Hfq, including the decoy effect exerted by CrcZ. On the other hand, the expression of GssA is influenced by various environmental factors, including glucose, and by the physiological state dictated by planktonic or aggregative growth, and this is integrated with the regulation by Hfq. Another key function subject to an Hfq/GssA interplay is pyocyanin production. As for glucose utilization and exotoxin A secretion, GssA plays a repressive role in pyocyanin secretion that can be influenced by Hfq, as described above. However, Hfq seems to play a stimulatory GssA-independent role in pyocyanin secretion. Therefore, Hfq can make a positive double input converge on the pathway of pyocyanin production, one independent of and the other dependent on GssA. Reciprocally, GssA can influence Hfq expression at both transcription and posttranscription levels, also depending on the type of growth, planktonic or aggregative. The influence of GssA on Hfq would also pass through CrcZ whose expression is repressed by GssA under some conditions, such as in the presence of glucose and succinate. This could strengthen the CCR exerted by succinate on nonpreferred carbon (CS) sources, but also that of glucose on the same sources. Finally, since cellular functions such as anoxic biofilm formation and anaerobic respiratory chain are regulated by Hfq with CrcZ-mediated modulation (25), GssA may also affect anoxic biofilm and anaerobic respiration in response to host glucose concentration.

Bacterial small RNAs (sRNAs) are key transducers of information from host environment sensors and are involved in the fine and coordinated regulation of bacterial pathogen virulence lifestyle genes (48–51). Of the large array of P. aeruginosa sRNAs, about 15 have been characterized, and a variety of virulence-linked functions were found to be under their control (12, 52). Hfq was shown to be directly involved in these regulations, acting as an RNA matchmaker between many of these sRNAs and their target mRNAs, or as a CrcZ-conditioned translational repressor via sequestration (12, 52). In this study, we explored a new sphere of the reciprocal relationship between Hfq and sRNAs highlighting the importance of the involvement of indirect interactions between Hfq and the sRNA GssA in the regulation of virulence factors and physiological functions in response to environmental stimuli and growth conditions. However, we assume that our results do not exclude that Hfq can assist GssA in interacting with target mRNAs or that GssA can compete with CrcZ and sequester Hfq under certain conditions such as that of anoxic biofilm where GssA has been seen abundantly bound to Hfq (25). As shown in the model of Fig. 12 (a more complete scheme of our results is depicted in Fig. S6), GssA responds extensively both to environmental signals and to physiological conditions related to the planktonic and aggregative growth forms. The evidence that the inactivation of several alternative σ factors dysregulates GssA levels is consistent with this wide variety of responses. The positive response to glucose and the negative transcriptional effect on 21 genes, both structural and regulatory, involved in glucose utilization strongly support the notion of an apical regulatory role of GssA in PA14 for exploiting this carbon source relevant in the human host. In PAO1, an Hfq knockout mutant is impaired in glucose utilization (53). We observed a similar effect in PA14. Given that Hfq could also positively regulate glucose genes independently, our results strongly indicate that Hfq and GssA interplay for the regulation of this carbon source utilization. We suggest that a key element of this interplay is the negative regulation exerted by Hfq on GssA. In PA14Δ*hfq* growing on glucose, GssA levels are strongly increased by the lack of Hfq and the presence of glucose. This in turn would enhance the repression of glucose genes and impair growth. We speculate that any regulation of Hfq is reflected in the utilization of glucose through GssA, together with other modulations of the expression of GssA itself independent of Hfq. We argue that this model is valid for target genes negatively regulated by GssA. Among these, there may be those involved in the secretion of exotoxin A, for which, from the comparison between the single mutants PA14Δ*gssA* and PA14Δ*hfq* and the double PA14Δ*hfq*Δ*gssA* mutants with PA14, we observe a pattern similar to that of glucose utilization. For the production of pyocyanin, our results also indicated an important negative role of GssA that can be influenced by Hfq as described above. However, Hfq seems to play a stimulatory GssA-independent role in pyocyanin production. Therefore, Hfq can make a positive double input converge on this pathway, one independent and the other dependent on GssA.

10.1128/mbio.02418-22.7 FIG S6 Schematic summary of the results of this work. Environmental inputs and physiological states dictated by the planktonic (green) or aggregative (orange) forms of growth that influence GssA expression either positively or negatively are indicated. For glucose, the stimulatory effect on GssA expression is independent of the form of growth. Hfq exerts a repressive role in GssA expression which in turn can influence GssA-regulated functions. Reciprocally, GssA influence Hfq expression at both the transcription and posttranscription levels, and also through the modulation of CrcZ expression. “T” and “P-T” indicate whether the evidence of regulation was observed at transcription or posttranscription levels, respectively. Green and orange spots indicate in which form of growth was observed the regulation. The asterisk indicates a specific negative role of GssA in influencing *hfq* mRNA levels when PA14 was grown statically in liquid BH. Download FIG S6, TIF file, 0.1 MB. Copyright © 2022 Santoro et al. 2022 Santoro et al. https://creativecommons.org/licenses/by/4.0/ This content is distributed under the terms of the Creative Commons Attribution 4.0 International license.

For the regulation of other functions such as the respiratory chain in anaerobiosis that strictly rely on *nuo* genes (40), GssA and Hfq could interplay by converging their positive regulation. However, a fraction of downregulation of *nuo* genes in our DEG analysis, which was performed in BHI, could be due to Hfq sequestration by increased levels of CrcZ in PA14Δ*gssA* in this medium. The latter effect might only be a part of a broader reciprocal influence of GssA on Hfq through CrcZ whose expression is repressed by GssA in some conditions such as in the presence of glucose and succinate. This could strengthen not only the CCR exerted by succinate on nonpreferred carbon (CS) sources but also that of glucose on the same sources. In addition, the influence of GssA on Hfq expression at both transcription and posttranscription levels could add further complexity to the GssA/Hfq interplay.

In summary, considering Hfq as a central regulatory hub in P. aeruginosa, our work has added a new spoke, the sRNA GssA, belonging to the accessory genome. GssA appears to be extensively linked to surrounding conditions including glucose, an important signal in the human host, and cell physiology, which could influence critical cellular functions under Hfq control such as CCR (23), anoxic biofilms, and the anaerobic respiratory chain (25). On the other hand, Hfq, through its repression of GssA, can modulate genes regulated by GssA, such as those for glucose utilization, exotoxin A secretion, and pyocyanin production. In addition to this, we imagine that many other genes are under Hfq-mediated regulation through a bridging role of GssA.

## MATERIALS AND METHODS

### Bacterial strains, plasmids, and culture conditions.

The bacterial strains and plasmids used in this study are listed in Table S3. Escherichia coli strains were routinely grown at 37°C in Luria-Bertani broth rich medium (LB). P. aeruginosa strains were grown at 37°C in LB, brain heart infusion rich medium (BHI), Pseudomonas isolation agar rich medium (PIA), Terrific Broth (TB), or M9 minimal medium supplemented with micronutrients and the indicated carbon sources. The added concentrations of antibiotics, carbon sources, and arabinose to induce the *P_BAD_* promoter of plasmid pGM931 are detailed in Text S1. The growth of planktonic cells with shaking (LSha) was performed by inoculating bacteria in liquid media at an OD_600_ of 0.1 in flasks or 15-mL tubes, followed by incubation with shaking at 120 rpm. Cell growth in the form of colony biofilms (CBio) was obtained by streaking or spreading bacterial cells on 1.5% agar plates. Growth of submerged cell aggregates (LSta) was performed by inoculating liquid media at an OD_600_ of 0.4 in flasks and incubating them statically for 48 h. Details on cell growth in anaerobiosis and the growth assays in the presence of different carbon sources on agar plates are provided in Text S1.

10.1128/mbio.02418-22.10 TABLE S3 Bacterial strains, plasmids, and oligonucleotides used in this study. (A) List of oligonucleotides. (B) List of bacterial strains and plasmids. Download Table S3, PDF file, 0.3 MB. Copyright © 2022 Santoro et al. 2022 Santoro et al. https://creativecommons.org/licenses/by/4.0/ This content is distributed under the terms of the Creative Commons Attribution 4.0 International license.

### Plasmid construction and mutant generation.

The construction of plasmids pGM-*gssA* and pGM-*gssA_GUGmut_* and the translational fusion *hfq*::*sfGFP* expressed by the pBBR1-MCS5 plasmid under the control of *P_LtetO-1_* is detailed in Text S1. PA14Δ*gssA* and PA14Δ*hfq*Δ*gssA* mutants were generated by allelic exchange using an enhanced method of markerless gene replacement with some modifications to adapt it to P. aeruginosa as described previously (36). Further details are provided in Text S1. The oligonucleotides used for plasmid construction and mutant generation are listed in Table S3.

### RNA isolation and analysis.

Total RNA from either P. aeruginosa aerobic cultures, anaerobic batch cultivations, or shift from aerobic to anaerobic conditions was prepared using 2 to 10 mL of bacterial cultures, as described previously (36). For total RNA preparation from CBio cultures, cells were collected from agar plates and treated as described previously (54). Total RNA from LSta conditions was prepared from 10-mL cultures incubated for 48 h. Treatment with terminator 5-phosphate-dependent exonuclease and primer extension analysis to identify GssA 5′ ends were performed as described previously (36). Northern blot analyses using γ-^32^P-labeled and biotinylated probes were performed as described previously (27, 54). Quantitative RT-PCR analysis (qRT-PCR) was performed as described previously (54, 55). qRT-PCRs were performed in triplicate from three independent biological replicates. Further details regarding the procedures used for RNA isolation and analysis are reported in Text S1 and for the oligonucleotides in Table S3.

### RNA sequencing and data analysis.

Total RNA for RNA-seq was extracted from PA14 and PA14Δ*gssA* grown in BHI medium at 37°C with shaking until OD_600_ of 2. For each strain, three independent biological replicates were performed. The samples of RNA were delivered to the company GalSeq for further processing, sequencing, and bioinformatics analysis. Specifications on RNA quality control and data analysis are reported in Text S1.

### sRNA/mRNA interaction *in vivo*.

Wild-type and Δ*gssA* strains were transformed with the pBBR1-*hfq*::*GFP* translational fusion coupled with either the pGM931 empty vector or the pGM-*gssA.* The pBBR1-*hfq*::*GFP* was also coupled with the pGM-*gssA_GUGmut_* plasmid in the wild-type strain. At least three independent clones were picked from every transformation and used in the setup of the experimental plan analyses. At least three independent biological replicates were performed for every experimental set. Bacterial cells were grown in LSha, LSta, or CBio conditions as described above. The quantification of the reporter sfGFP activity was performed as described previously (54) and is detailed in Text S1.

### Quantification of pyocyanin and exotoxin A and performance of the Congo red binding assay.

The procedures for the quantitative analysis of pyocyanin levels, detection of exotoxin A by Western blotting, and Congo red binding assay are detailed in Text S1.

### Statistical analysis.

Statistical analyses were performed with GraphPad Prism 6. Results are presented as means ± the standard deviations (SD). Significance, determined using one-way analysis of variance (ANOVA) with *post hoc* Tukey’s honestly significant difference [HSD], is indicated in the figures by asterisks (*, *P* < 0.05; **, *P* < 0.01; ***, *P* < 0.001; ****, *P* < 0.0001).
