# Supplementary figures and images for: Multifaceted Interplay between Hfq and the Small RNA GssA in Pseudomonas aeruginosa
Source: mBio. 2022 Dec 8;14(1):e02418-22. doi: 10.1128/mbio.02418-22 (PMC9973299; doi:10.1128/mbio.02418-22)

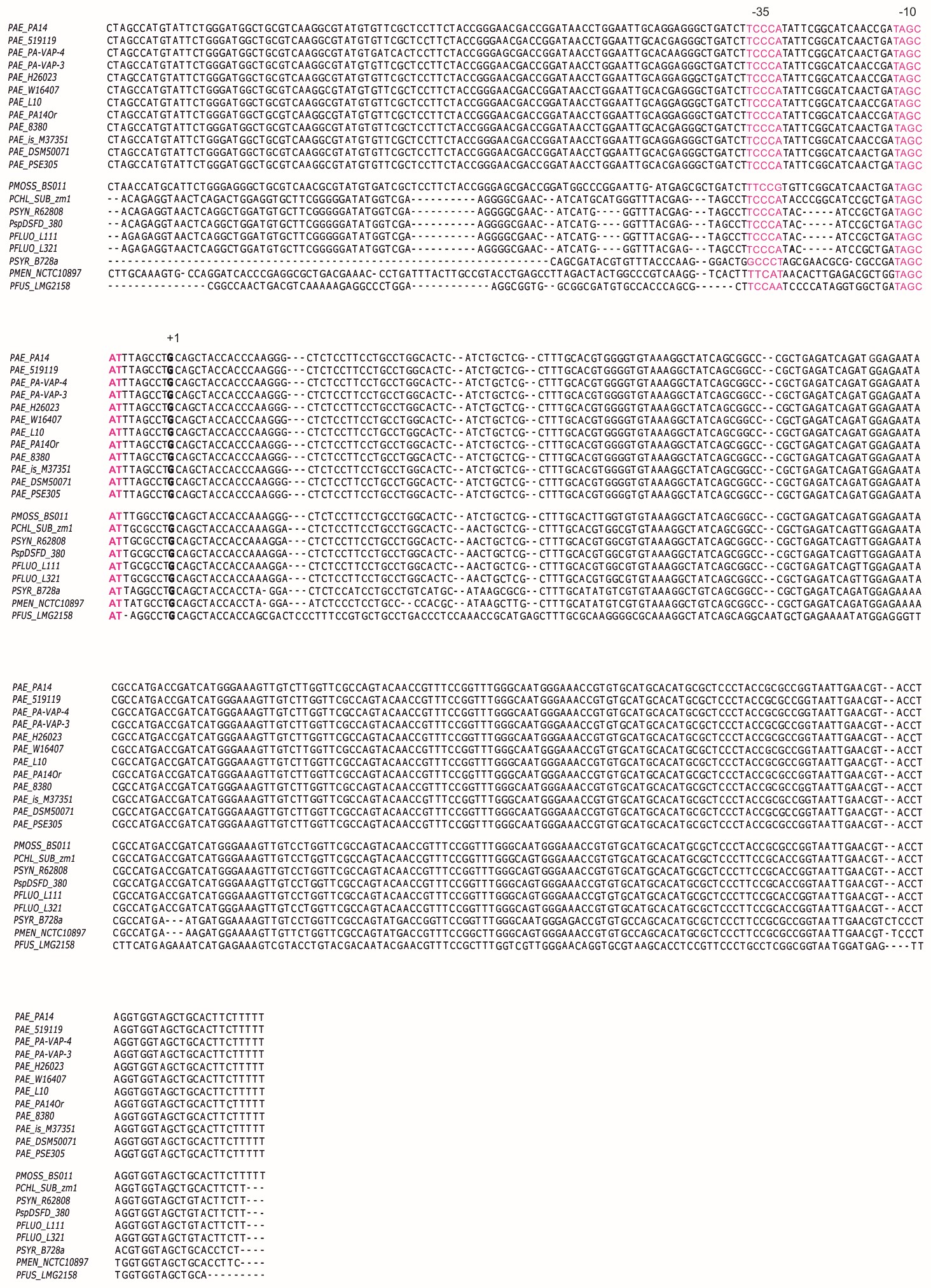

Supplement: FIG S1 [file mbio.02418-22-sf001.jpg]

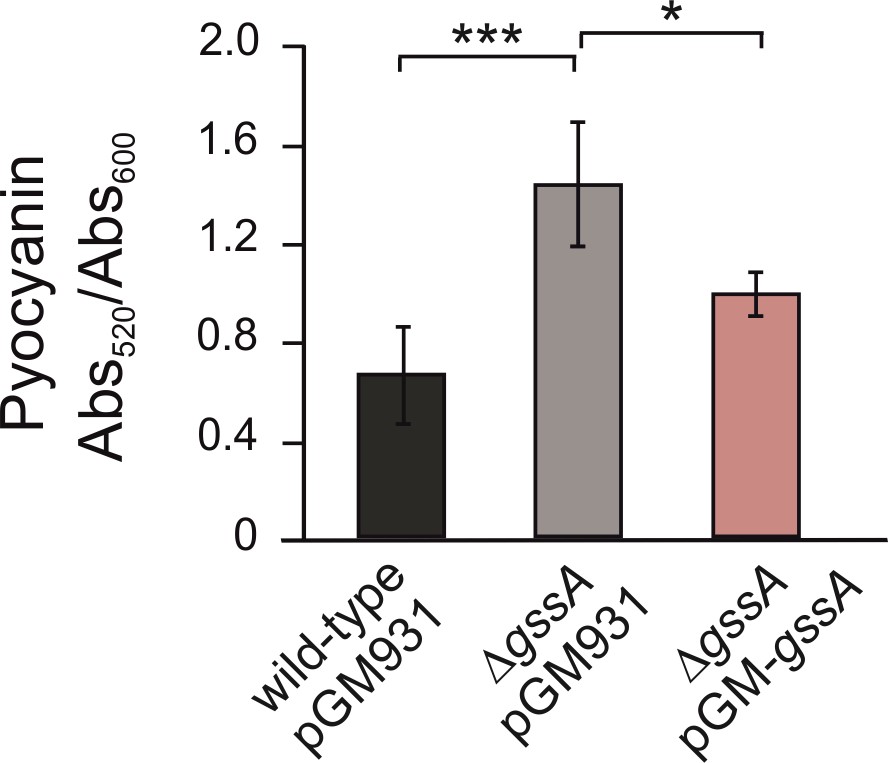

Supplement: FIG S2 [file mbio.02418-22-sf002.jpg]

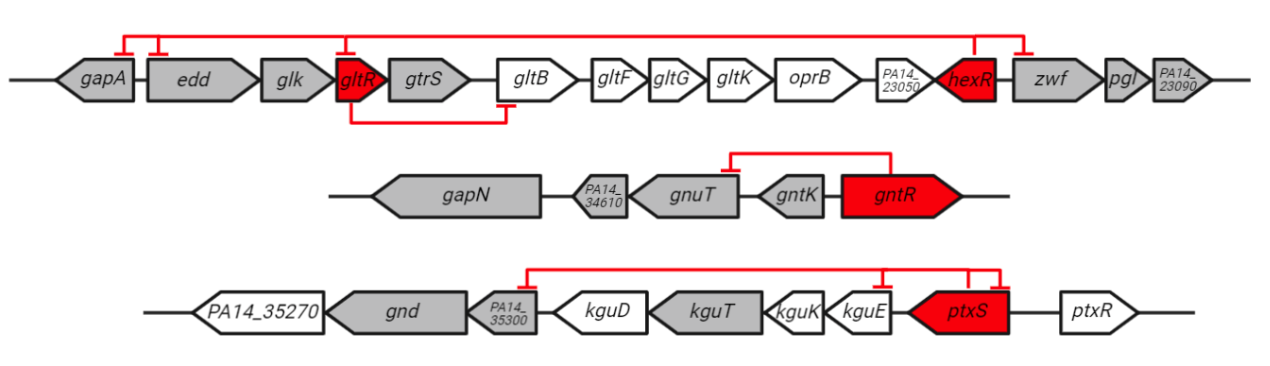

Supplement: FIG S3 [file mbio.02418-22-sf003.tif]

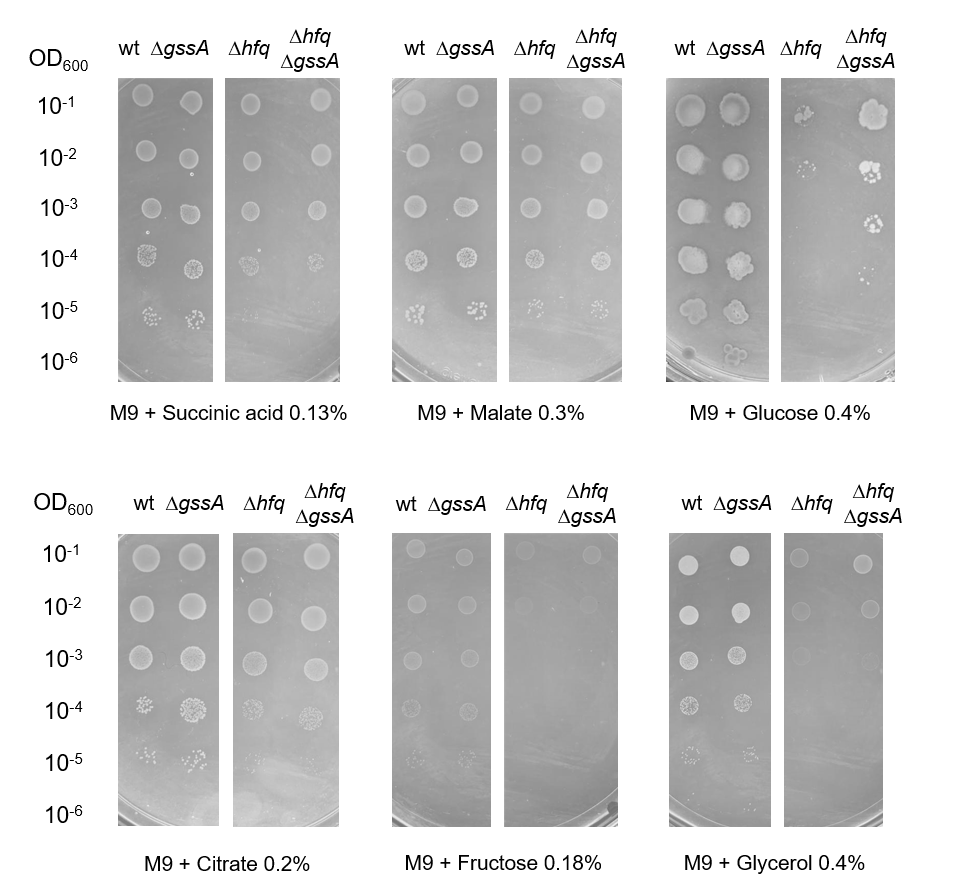

Supplement: FIG S4 [file mbio.02418-22-sf004.tif]

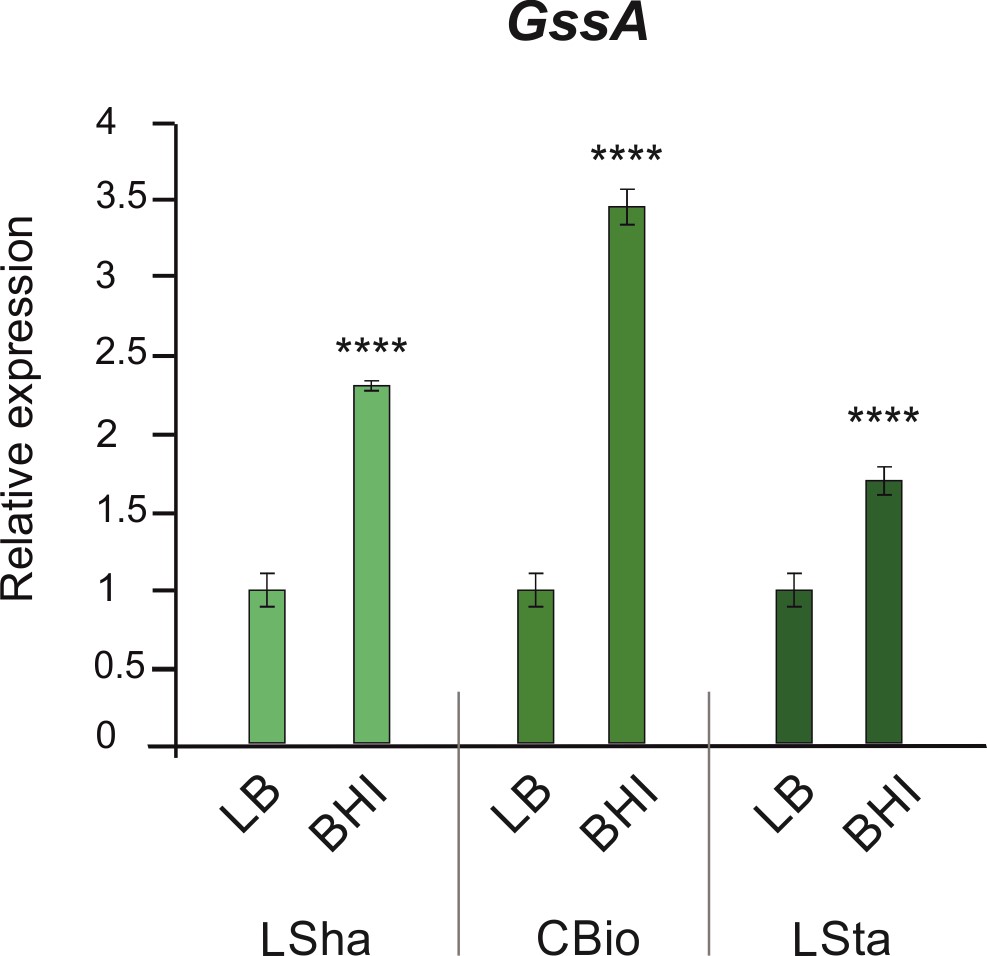

Supplement: FIG S5 [file mbio.02418-22-sf005.jpg]

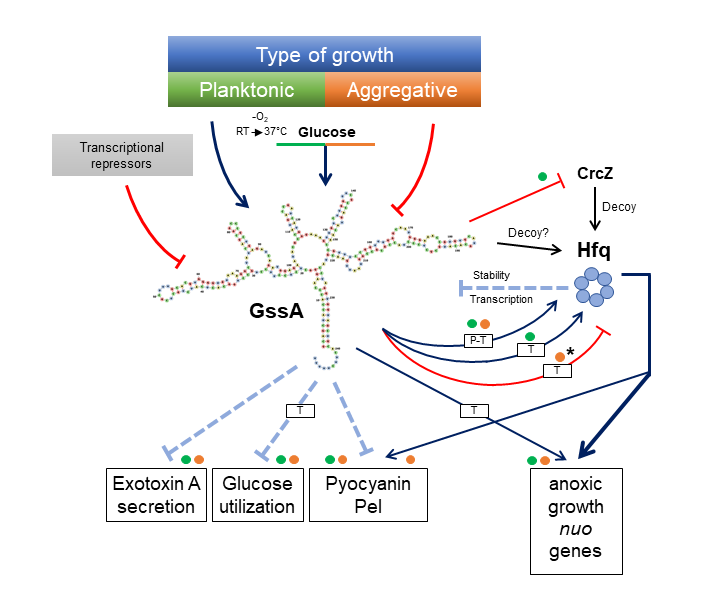

Supplement: FIG S6 [file mbio.02418-22-sf006.tif]
