# Supplementary material for: Multifaceted Interplay between Hfq and the Small RNA GssA in Pseudomonas aeruginosa
Source: mBio. 2022 Dec 8;14(1):e02418-22. doi: 10.1128/mbio.02418-22 (PMC9973299; doi:10.1128/mbio.02418-22)
Supplement: TABLE S1 [file mbio.02418-22-st001.pdf]

TABLE S1 Lists of transcriptional regulators under either direct or indirect modulation of a panel of alternative sigma factors

RpoS

		Δ*rpoS* vs. WT*			ChIP seq analysis*		
**PA14 ID**	**Gene name**	**log2 fold change of gene expression**	**P value**	**P value (corrected)**	**Enrichment of promoter regions by ChIP-seq**	**Product name**	**PseudoCAP category**
PA14_02260	*cheY*	-4,491	0,000	0,000	N/A	putative two-component response regulator	Chemotaxis; Adaptation / Protection; Two-component regulatory systems
PA14_04270		-2,205	0,000	0,003	N/A	putative transcriptional regulator	Transcriptional regulators
PA14_06770	*nirQ*	2,567	0,000	0,000	N/A	regulatory protein NirQ	Energy metabolism; Central intermediary metabolism
PA14_13150		-1,658	0,000	0,000	N/A	putative transcriptional regulator	Transcriptional regulators
PA14_17480	*rpoS*	-8,078	0,000	0,000	N/A	sigma factor RpoS	Transcriptional regulators
PA14_28130		2,567	0,000	0,003	N/A	putative transcriptional regulator	Transcriptional regulators
PA14_29730		-1,984	0,000	0,003	N/A	putative two-component response regulator	Transcriptional regulators; Two-component regulatory systems
PA14_30830		-4,229	0,000	0,000	N/A	putative two-component response regulator	Transcriptional regulators; Two-component regulatory systems
PA14_50220	*fleQ*	-1,068	0,001	0,015	N/A	transcriptional regulator FleQ	Motility / Attachment; Transcriptional regulators
PA14_55810		-2,630	0,000	0,000	N/A	putative two-component response regulator	Transcriptional regulators; Two-component regulatory systems
PA14_63210		-4,206	0,000	0,000	N/A	putative two-component response regulator	Two-component regulatory systems; Transcriptional regulators; Motility / Attachment; Cell wall / LPS / capsule
PA14_66850	*phaD*	-3,257	0,000	0,000	N/A	putative transcriptional regulator, TetR family	Transcriptional regulators
PA14_69750		-1,709	0,002	0,021	N/A	putative transcriptional regulator	Transcriptional regulators
PA14_71640		-1,599	0,006	0,045	N/A	putative transcriptional regulator, LysR family	Transcriptional regulators
PA14_71780		-1,200	0,007	0,050	N/A	putative transcriptional regulator, RpiR family	Transcriptional regulators
PA14_02260	*cheY*	N/A	N/A	N/A	yes	putative two-component response regulator	Chemotaxis; Adaptation / Protection; Two-component regulatory systems
PA14_04820		N/A	N/A	N/A	yes	putative transcriptional regulator, TetR family	Transcriptional regulators
PA14_17380		N/A	N/A	N/A	yes	putative transcriptional regulator, LysR family	Transcriptional regulators
PA14_17790		N/A	N/A	N/A	yes	putative LysR-family transcriptional regulator	Transcriptional regulators
PA14_21850		N/A	N/A	N/A	yes	putative transcriptional regulator	Transcriptional regulators
PA14_23590		N/A	N/A	N/A	yes	putative transcriptional regulator	Transcriptional regulators
PA14_26140	*cifR*	N/A	N/A	N/A	yes	putative transcriptional regulator	Transcriptional regulators
PA14_28130		N/A	N/A	N/A	yes	putative transcriptional regulator	Transcriptional regulators
PA14_30830		N/A	N/A	N/A	yes	putative two-component response regulator	Transcriptional regulators; Two-component regulatory systems
PA14_31780		N/A	N/A	N/A	yes	putative transcriptional regulator, LysR family	Transcriptional regulators
PA14_36300		N/A	N/A	N/A	yes	putative transcriptional regulator, TetR family	Transcriptional regulators
PA14_39980	*qscR*	N/A	N/A	N/A	yes	probable transcriptional regulator	Transcriptional regulators
PA14_40440		N/A	N/A	N/A	yes	putative transcriptional regulator, LysR family	Transcriptional regulators
PA14_41260		N/A	N/A	N/A	yes	putative two-component response regulator	Transcriptional regulators; Two-component regulatory systems
PA14_41810		N/A	N/A	N/A	yes	putative transcriptional regulator	Transcriptional regulators
PA14_43770		N/A	N/A	N/A	yes	putative transcriptional regulator	Transcriptional regulators
PA14_47520		N/A	N/A	N/A	yes	putative transcriptional regulator	Transcriptional regulators
PA14_49790		N/A	N/A	N/A	yes	probable transcriptional regulator	Transcriptional regulators
PA14_55810		N/A	N/A	N/A	yes	putative two-component response regulator	Transcriptional regulators; Two-component regulatory systems
PA14_63210		N/A	N/A	N/A	yes	putative two-component response regulator	Two-component regulatory systems; Transcriptional regulators; Motility / Attachment; Cell wall / LPS / capsule
PA14_69750		N/A	N/A	N/A	yes	putative transcriptional regulator	Transcriptional regulators
PA14_70560		N/A	N/A	N/A	yes	putative transcriptional regulator, LysR family	Transcriptional regulators

* Analyses published by Schulz et al. 2015 https://doi.org/10.1371/journal.ppat.1004744

TABLE S1 Lists of transcriptional regulators under either direct or indirect modulation of a panel of alternative sigma factors

SigX

		Δ*sigX* vs. WT* (low salt, equal mock induction period)			Δ*sigX* vs. WT* (low salt, equal OD when harvesting)			ChIP seq analysis*		
**PA14 ID***	**Gene name**	**log2 fold change of gene expression**	**P value**	**P value (corrected)**	**log2 fold change of gene expression**	**P value**	**P value (corrected)**	**Enrichment of promoter regions by ChIP-seq**	**Product name**	**PseudoCAP category**
PA14_00600		999,000	0,000	0,004	5,696	0,000	0,000	N/A	putative transcriptional regulator	Transcriptional regulators
PA14_04270		1,044	0,147	0,368	1,865	0,000	0,004	N/A	putative transcriptional regulator	Transcriptional regulators
PA14_07950	*prtN*	3,575	0,000	0,000	3,261	0,000	0,000	N/A	transcriptional regulator PrtN	Transcriptional regulators
PA14_08630		-0,979	0,053	0,203	-1,193	0,016	0,049	N/A	putative transcriptional regulator	Transcriptional regulators
PA14_08710	*nusG*	-0,920	0,002	0,020	-1,739	0,000	0,001	N/A	transcription antitermination protein NusG	Transcription, RNA processing and degradation
PA14_10290	*acoR*	2,352	0,000	0,005	2,724	0,000	0,000	N/A	transcriptional regulator AcoR	Transcriptional regulators
PA14_13150		1,864	0,000	0,004	1,595	0,002	0,013	N/A	putative transcriptional regulator	Transcriptional regulators
PA14_19850		1,184	0,373	0,591	3,363	0,000	0,003	N/A	putative transcriptional regulator	Transcriptional regulators
PA14_26140	*cifR*	0,000	1,000	1,000	1,519	0,016	0,048	N/A	putative transcriptional regulator	Transcriptional regulators
PA14_28130		2,628	0,117	0,326	3,186	0,000	0,000	N/A	putative transcriptional regulator	Transcriptional regulators
PA14_32190		6,028	0,004	0,030	4,108	0,000	0,000	N/A	putative transcriptional regulator	Transcriptional regulators
PA14_33260	*pvdS*	999,000	0,008	0,050	999,000	0,006	0,025	N/A	sigma factor PvdS	Transcriptional regulators
PA14_36300		-1,968	0,002	0,016	-1,409	0,003	0,016	N/A	putative transcriptional regulator, TetR family	Transcriptional regulators
PA14_37080		-2,093	0,009	0,058	-2,166	0,003	0,016	N/A	putative transcriptional regulator	Transcriptional regulators
PA14_42060		0,000	1,000	1,000	1,665	0,015	0,047	N/A	putative transcriptional regulator	Transcriptional regulators
PA14_43820		0,000	1,000	1,000	1,708	0,008	0,031	N/A	putative transcriptional regulator	Transcriptional regulators
PA14_47910		3,540	0,046	0,182	3,915	0,003	0,016	N/A	putative transcriptional regulator	Transcriptional regulators
PA14_50220	*fleQ*	-0,953	0,003	0,026	-1,400	0,002	0,012	N/A	transcriptional regulator FleQ	Motility / Attachment; Transcriptional regulators
PA14_53730		1,554	0,001	0,006	1,776	0,002	0,012	N/A	probable transcriptional regulator	Transcriptional regulators
PA14_56620		2,285	0,011	0,066	1,539	0,010	0,038	N/A	putative transcriptional regulator	Transcriptional regulators
PA14_62490	*dksA*	-1,080	0,000	0,004	-1,916	0,000	0,002	N/A	suppressor protein DksA	Transcriptional regulators; Adaptation / Protection; DNA replication, recombination, modification and repair
PA14_72650		-2,142	0,001	0,007	-2,686	0,000	0,000	N/A	putative transcriptional regulator	Transcriptional regulators
PA14_02390		N/A	N/A	N/A	N/A	N/A	N/A	yes	putative transcriptional regulator	Transcriptional regulators
PA14_03070	*pobR*	N/A	N/A	N/A	N/A	N/A	N/A	yes	putative transcriptional regulator	Transcriptional regulators
PA14_09960		N/A	N/A	N/A	N/A	N/A	N/A	yes	putative transcriptional regulator	Transcriptional regulators
PA14_16350		N/A	N/A	N/A	N/A	N/A	N/A	yes	putative two-component response regulator	Transcriptional regulators; Two-component regulatory systems
PA14_24710		N/A	N/A	N/A	N/A	N/A	N/A	yes	putative two-component response regulator	Transcriptional regulators; Two-component regulatory systems
PA14_29300		N/A	N/A	N/A	N/A	N/A	N/A	yes	putative transcriptional regulator	Transcriptional regulators
PA14_29590		N/A	N/A	N/A	N/A	N/A	N/A	yes	putative transcriptional regulator	Transcriptional regulators
PA14_29620	*norR*	N/A	N/A	N/A	N/A	N/A	N/A	yes	putative transcriptional regulator	Transcriptional regulators; Adaptation / Protection
PA14_30970	*bphR*	N/A	N/A	N/A	N/A	N/A	N/A	yes	putative transcriptional regulator	Transcriptional regulators
PA14_31560		N/A	N/A	N/A	N/A	N/A	N/A	yes	putative transcriptional regulator, LysR family	Transcriptional regulators
PA14_40550		N/A	N/A	N/A	N/A	N/A	N/A	yes	putative transcriptional regulator, LysR family	Transcriptional regulators
PA14_46570		N/A	N/A	N/A	N/A	N/A	N/A	yes	putative transcriptional regulator	Transcriptional regulators
PA14_46850		N/A	N/A	N/A	N/A	N/A	N/A	yes	putative transcriptional regulator	Transcriptional regulators
PA14_48190		N/A	N/A	N/A	N/A	N/A	N/A	yes	putative transcriptional regulator	Transcriptional regulators
PA14_52070		N/A	N/A	N/A	N/A	N/A	N/A	yes	probable transcriptional regulator	Transcriptional regulators
PA14_53920		N/A	N/A	N/A	N/A	N/A	N/A	yes	probable transcriptional regulator	Transcriptional regulators
PA14_64910		N/A	N/A	N/A	N/A	N/A	N/A	yes	putative transcriptional regulator, LysR family	Transcriptional regulators
PA14_71640		N/A	N/A	N/A	N/A	N/A	N/A	yes	putative transcriptional regulator, LysR family	Transcriptional regulators

* Analyses published by Schulz et al. 2015 https://doi.org/10.1371/journal.ppat.1004744

TABLE S1 Lists of transcriptional regulators under either direct or indirect modulation of a panel of alternative sigma factors

PvdS

		Δ*pvdS* vs. WT*			ChIP seq analysis*		
**PA14 ID***	**Gene name**	**log2 fold change of gene expression**	**P value**	**P value (corrected)**	**promoter regions by ChIP-seq**	**Product name**	**PseudoCAP category**
PA14_06970		1,559	0,000	0,018	N/A	putative transcriptional regulator, Cro/CI family	Transcriptional regulators
PA14_33260	*pvdS*	-2,331	0,000	0,000	N/A	sigma factor PvdS	Transcriptional regulators
PA14_38380	*amrR*	1,285	0,001	0,041	N/A	putative transcriptional regulator	Transcriptional regulators
PA14_42390	*exsA*	1,874	0,000	0,001	N/A	transcriptional regulator ExsA	Protein secretion / Export apparatus; Transcriptional regulators

* Analyses published by Schulz et al. 2015 https://doi.org/10.1371/journal.ppat.1004744

TABLE S1 Lists of transcriptional regulators under either direct or indirect modulation of a panel of alternative sigma factors

AlgU

		Δ*algU* vs. WT*			ChIP seq analysis*		
PA14 ID	Gene name	log2 fold change of gene expression	P value	P value (corrected)	Enrichment of promoter regions by ChIP-seq	Product name	PseudoCAP category
PA14_22760	*cpxR*	-3,298	0,000	0,002	N/A	putative transcriptional regulator in 2-component system	Transcriptional regulators; Two-component regulatory systems
PA14_33440		-2,718	0,000	0,012	N/A	putative transcriptional regulator, LysR family	Transcriptional regulators
PA14_35070		-3,711	0,001	0,027	N/A	putative transcriptional regulator, AraC family	Transcriptional regulators
PA14_35380	*ptxR*	-2,650	0,002	0,046	N/A	transcriptional regulator PtxR	Transcriptional regulators
PA14_54430	*algU*	-7,996	0,000	0,000	N/A	sigma factor AlgU	Transcriptional regulators
PA14_72380	*algB*	-4,664	0,000	0,000	N/A	two-component response regulator AlgB	Transcriptional regulators; Two-component regulatory systems
PA14_72390	*kinB*	-4,032	0,000	0,000	N/A	putative two-component sensor	Two-component regulatory systems
PA14_04930	*rpoH*	N/A	N/A	N/A	yes	RNA polymerase sigma-32 factor	Transcriptional regulators
PA14_10830		N/A	N/A	N/A	yes	putative transcriptional regulator, LysR family	Transcriptional regulators
PA14_16380		N/A	N/A	N/A	yes	putative LysR-family transcriptional regulator	Transcriptional regulators
PA14_18080		N/A	N/A	N/A	yes	putative transcriptional regulator, TetR family	Transcriptional regulators; Antibiotic resistance and susceptibility
PA14_19380		N/A	N/A	N/A	yes	putative transcriptional regulator	Transcriptional regulators
PA14_27940		N/A	N/A	N/A	yes	putative two-component response regulator	Transcriptional regulators; Two-component regulatory systems
PA14_32460		N/A	N/A	N/A	yes	putative transcriptional regulator	Transcriptional regulators
PA14_41575	*sigX*	N/A	N/A	N/A	yes	ECF sigma factor SigX	Transcriptional regulators
PA14_44780		N/A	N/A	N/A	yes	putative transcriptional regulator	Transcriptional regulators
PA14_52920		N/A	N/A	N/A	yes	probable transcriptional regulator	Transcriptional regulators
PA14_54420	*mucA*	N/A	N/A	N/A	yes	anti-sigma factor MucA	Secreted factors (toxins, enzymes, alginate); Transcriptional regulators; Cell wall / LPS / capsule
PA14_54430	*algU*	N/A	N/A	N/A	yes	sigma factor AlgU	Transcriptional regulators
PA14_58380		N/A	N/A	N/A	yes	putative transcriptional regulator	Transcriptional regulators
PA14_64050		N/A	N/A	N/A	yes	putative two-component response regulator	Transcriptional regulators; Two-component regulatory systems
PA14_68420		N/A	N/A	N/A	yes	putative transcriptional regulator, LysR family	Transcriptional regulators

* Analyses published by Schulz et al. 2015 https://doi.org/10.1371/journal.ppat.1004744

TABLE S1 Lists of transcriptional regulators under either direct or indirect modulation of a panel of alternative sigma factors

FpvI

		Δ*fpvI* vs. WT*			ChIP seq analysis*		
**PA14 ID***	**Gene name**	**log2 fold change of gene expression**	**P value**	**P value (corrected)**	**promoter regions by ChIP-seq**	**Product name**	**PseudoCAP category**
PA14_31560		0,939	0,049	1,000	N/A	putative transcriptional regulator, LysR family	Transcriptional regulators
PA14_33800	*fpvI*	-1,237	0,011	1,000	N/A	putative sigma-7 factor, ECF subfamily	Transcriptional regulators; Transport of small molecules
PA14_47520		-1,185	0,027	1,000	N/A	putative transcriptional regulator	Transcriptional regulators
PA14_04270		N/A	N/A	N/A	N/A	putative transcriptional regulator	Transcriptional regulators

* Analyses published by Schulz et al. 2015 https://doi.org/10.1371/journal.ppat.1004744
