# Supplementary material for: Multifaceted Interplay between Hfq and the Small RNA GssA in Pseudomonas aeruginosa
Source: mBio. 2022 Dec 8;14(1):e02418-22. doi: 10.1128/mbio.02418-22 (PMC9973299; doi:10.1128/mbio.02418-22)
Supplement: TEXT S1 [file mbio.02418-22-s0001.pdf]

Multifaceted interplay between Hfq and the small RNA GssA in Pseudomonas aeruginosa

Silvia Santoro, Costanza Paganin, Sara Gilardi, Tarcisio Brignoli, Giovanni Bertoni* and Silvia Ferrara*

Department of Biosciences, Università degli Studi di Milano, Milan, Italy

*Author to whom correspondence should be addressed.

silvia.ferrara@unimi.it

giovanni.bertoni@unimi.it

Supplemental file with detailed Materials and Methods

Bacterial strains and culture conditions

E. coli strains were routinely grown at 37°C in Luria-Bertani Broth rich medium (LB). *P. aeruginosa* strains were grown at 37°C in LB, Brain Heart Infusion rich medium (BHI), Pseudomonas isolation agar rich medium (PIA), Terrific Broth (TB), or M9 minimal medium supplemented with micronutrients (1) and the indicated carbon sources. For selective *E. coli* growth, antibiotics were added at the following concentrations: 20 µg/ml gentamicin, 100 µg/ml ampicillin, 50 µg/ml chloramphenicol, and 50 µg/ml kanamycin; for selective *P. aeruginosa* growth, gentamicin and carbenicillin were added at 60 and 300 µg/ml, respectively. For *Δhfq P. aeruginosa* mutant strains, gentamicin and carbenicillin were added to 30 and 150 µg/ml, respectively. To induce the P_{BAD} promoter of plasmid pGM931, arabinose was added to the growth media at a final concentration of 10 mM.

The growth of planktonic cells with shaking (LSha) was performed as follows. Liquid media were inoculated at an OD_{600} of 0.1 in flasks or 15-ml tubes and incubated with shaking at 120 rpm. Planktonic cell growth in anaerobiosis and the shift from aerobic to anaerobic conditions were performed with a stirring speed of 500 rpm in an 800 ml-Biostat-Q system bioreactor (B-Braun) as described previously (2). Cell growth in form of colony biofilm (CBio) was obtained by streaking or spreading bacterial cells on 1.5% agar plates. Growth of submerged cell aggregates (LSta) was performed by

inoculating liquid media at an OD_{600} of 0.4 in flasks and incubating them statically for 48 hrs.

For the growth assays in presence of different carbon sources on agar plates, overnight liquid cultures grown in LB were washed twice, normalized to an OD_{600} of 1 (corresponding to 1×10^9 CFU/ml), and serially diluted until 10^{-6}. 2 µl of each dilution were spotted and plates were incubated at 37°C. Growth was monitored daily. Carbon sources were supplied to LB or M9 agar plates at the following concentrations: glucose 22.2 mM (0.4%), citrate 7 mM (0.2%), fructose 10 mM (0.18%), glycerol 40 mM (0.4%), malate 20 mM (0.3%), succinate 8 mM (0.13%). To test the effects of oxygen starvation, the anaerobic atmosphere was induced in a jar from an Oxoid AnaeroGen sachet. The Oxoid Anaerobic indicator provided visual control of the achievement and maintenance of anaerobic conditions.

Plasmid construction and mutant generation

To construct plasmid pGM-*gssA* and pGM-*gssA*$_{GUGmut}$, the *gssA* gene was PCR-amplified from PA14 genomic DNA with oligo pairs 9/10 and 9/11, respectively, digested with NcoI-PstI and cloned into the pGM931 vector under the arabinose-inducible P_{BAD} promoter (3). The cloned sequences were screened using oligos 23/24. PA14*ΔgssA* and PA14*ΔhfqΔgssA* mutants were generated by allelic exchange using an enhanced method of markerless gene replacement as described in (4) with some modifications to adapt it to *P. aeruginosa* as described previously (2). To construct PA14Δ*gssA*, a deletion from −36 to +237 from the *gssA* transcription start site was generated as follows. The TS1 region spanning the left 537-bp flanking sequence of *gssA* was amplified with oligo pair 12/13. The TS2 region spanning the right 498-bp flanking sequence of *gssA* was amplified with oligos 14/15. SOE-PCR with oligos 12 and 15 was performed to join TS1 and TS2. The joined TS1-TS2 DNA fragments were digested Sac*I*-Bam*HI* and cloned in *E. coli* CC118 λpir into the delivery vector pSEVA612S. Clones were screened using oligo pairs 25/26. The procedure of gene transfer and selection of the *P. aeruginosa* clones with allelic exchange was described previously (2). The screening of the clones carrying the deleted *gssA* gene was performed with oligos 16/17and mutants were checked by Northern blot. The double mutant strain PA14*Δhfq*Δ*gssA* was generated in the PA14Δ*gssA* genetic background

through the additional deletion of the *hfq* gene. All plasmid constructs and deletion mutants were checked by sequencing.

Translational fusion *hfq::sfGFP* expressed by the pBBR1-MCS5 plasmid under the control of $P_{LtetO-1}$ was constructed as follows. Oligo pairs 18/19 were used to generate a DNA fragment spanning −464 nt from *hfq* translation start site and the first +36 codons of *hfq* mRNA. The fragment was digested NsiI-NheI and cloned into the sfGFP reporter vector pXG10-SF (5) giving rise to plasmid pXG10-*hfq::sfGFP*. After a check by sequencing using oligo 22, the DNA fragment spanning $P_{LtetO-1}$ and the sfGFP fusions was then amplified from pXG10-*hfq::sfGFP* with oligos 20/21, digested with ClaI/XbaI, and cloned into pBBR1-MCS5. The screening was performed by PCR using oligos M13 (27/28) and checked by sequencing.

RNA isolation and analysis

Bacterial cultures were grown overnight or until the indicated OD_{600} in liquid with shaking or stirring (LSha), overnight on the surface of medium-agar (CBio), or for 48 hrs in liquid statically (LSta). For LSha and LSta, cells were directly separated from the growth medium by centrifugation. For CBio, samples of cells were collected from the agar surface by inoculation loops, resuspended in PBS buffer, and then pelleted by centrifugation. Total RNA from cells was prepared as described previously (2, 6, 7). Pelleted cells were resuspended in RNAprotect Cell Reagent (Qiagen), incubated for 5 min at room temperature, pelleted by centrifugation, and stored at −80°C until use. Total RNA extraction was performed by the RNeasy Mini Kits (Qiagen) according to the manufacturer’s instructions, including RNase-free DNaseI in-column treatment. The concentration and quality of the extracted RNAs were assessed by a Biospectrometer (Eppendorf). RNA integrity was checked by electrophoresis of samples on denaturing (7 M urea) 6% polyacrylamide gel (dPAGE).

Primer extension analysis to identify GssA 5' ends was performed as described in (2) using total RNA extracted from PA14 cells grown in LSha at 37°C and 0.5 pmoles of radio-labeled oligo 5. Sanger sequencing reactions performed with radio-labeled oligo 8 on purified *fusA1* DNA amplified with oligos 6/7 were used as molecular weight markers.

Treatment with terminator 5-phosphate-dependent exonuclease was performed in terminator reaction buffer A (Epicentre) according to the manufacturer’s instructions.

Northern blot analyses using γ-^{32}P labeled probes were performed as described previously (6). Oligos 1 and 3 were used to probe GssA and 5S, respectively. For Northern blot analyses using biotinylated probes, hybridizations were performed using the North2South Chemiluminescent Hybridization and Detection kit (Thermo Scientific) as described previously (7). Biotinylated oligos 2 and 4 were used to probe GssA and 5S, respectively.

Quantitative RT-PCR analysis (qRT-PCR) was performed as described previously (7). cDNAs were synthesized from 3 µg of total purified RNA using Superscript III Reverse Transcriptase (Invitrogen) according to the manufacturer's instructions. qRT-PCR was performed in triplicate using TB green (Takara) PCR Master Mix on a CFX Connect Real-Time System (Bio-Rad). Oligo pairs were used for amplification as follows. 29/30 for 16S (9), 31/32 for *gssA*, 33/34 for *ptxS*, 43/44 for *hfq*, and 45/46 for CrcZ. Oligo pairs 35/36, 37/38, 39/40, and 41/42 were modified from (8) and were used for amplification of *ptxR*, *toxA*, *regA*/*toxR*, and *pvcB*, respectively. The reaction procedure involved incubation at 95°C for 5 min and 39 cycles of amplification at 95°C for 15 s, 58°C for 20 s, and 72.5°C for 30 s. The calculation of the relative expression (9) of the analyzed genes in each strain was performed by normalizing mRNA amounts to 16S ribosome RNA (ΔC_T). For the overall comparison of mRNA levels graphs were plotted as $2^{-\Delta C_T}$. For the comparison with a reference strain/condition, the results were plotted as $2^{-\Delta\Delta C_T}$ after relating the ΔC_T in the analyzed condition to the reference strain/condition ($\Delta\Delta C_T$).

RNA sequencing and data analysis

Total RNA for RNA-seq was extracted from PA14 and PA14Δ*gssA* grown in BHI medium at 37 °C with shaking until OD_{600} of 2. For each strain, three independent biological replicates were performed. The samples of RNA were delivered to the company GalSeq (www.galseq.com) for further processing, sequencing, and bioinformatics analysis. The concentration and the purity of the RNA starting material were determined spectrophotometrically while RNA Integrity Number (RIN) was measured on an Agilent TapeStation 4200. An additional DNase step was included to cleave DNA that could interfere with rRNA removal and negatively affect library preparation and sequencing. RNA libraries were generated starting from 3 µg of total RNA. rRNA was depleted with Illumina Ribo-Zero rRNA Removal Kit (Gram-Negative

Bacteria) and libraries were generated according to Illumina TruSeq® Stranded mRNA Library Prep protocol. RNA libraries were accurately quantified with a fluorometric method: QUBIT dsDNA HS assay kit and the size and purity were verified on an Agilent TapeStation 4200. RNA libraries were then sequenced on an Illumina HiSeq with paired-end reads 150 bp long. For RNA-Seq data analysis, *P. aeruginosa* UCBPP-PA14 (NCBI: NC_008463.1) genome and annotation (GTF) were downloaded from the Pseudomonas Genome Database. Alignment of paired Fastq RNA-Seq reads to *P. aeruginosa* UCBPP-PA14 genome was performed using the STAR aligner (v2.7.0) (10) with the quantMode TranscriptomeSAM GeneCounts option turned on. SAM files were converted to BAM, sorted, and indexed using SAMtools (11). Differential gene expression analysis was performed using DESeq2 (12) using a design = ~ condition model and dedicated R scripts. The false discovery rate was controlled using the Benjamini-Hochberg procedure, with an adjusted p-value (FDR) ≤ 0.1. *P. aeruginosa* gene IDs were annotated with a gene description field using a custom tool (IdToGeneNamesScript) using the *P. aeruginosa* UCBPP-PA14 GTF file as annotation input. RNA-seq data have been deposited in the ArrayExpress database at EMBL-EBI under the accession number E-MTAB-12073.

sRNA/mRNA interaction *in vivo*

Wild-type and Δ*gssA* strains were transformed with the pBBR1-*hfq::GFP* translational fusion coupled with either the pGM931 empty vector or the pGM-*gssA.* The pBBR1-*hfq::GFP* was also coupled with the pGM-$gssA_{GUGmut}$ plasmid in the wild-type strain. At least three independent clones were picked from every transformation and used in the setup of the experimental plan analyses. At least three independent biological replicates were performed for every experimental set. Bacterial cells were grown in LSha, LSta, or Cbio conditions as described above. Cell samples were pelleted and resuspended in PBS (10 mM Na_3PO_4, 150 mM NaCl). Starting from an OD_{600} of 1, samples were serially diluted 1.33-fold to OD_{600} of 0.75, 0.5, and 0.25, respectively, and aliquots of 200 µl were transferred to black polystyrene 96-well microplates with a clear, flat bottom (Corning). The absorbance (Abs_{595}) and fluorescence intensity ($FI_{485/535}$) were measured in an EnSight Multimode Plate Reader (PerkinElmer) using Kaleido data acquiring software. GFP activity was expressed in arbitrary units (AU) as $FI_{485/535}/Abs_{595}$.

Pyocyanin quantification assay

Pyocyanin production was assayed as described in (13). 5 ml of medium supernatants from cultures grown in LSha or LSta conditions were added with 3 ml of chloroform, vortexed for 20 sec, and centrifuged for 10 min at 3000 x rpm. The bottom phase was collected and added with 1 ml of 0.2 M HCl. The samples were vortexed for 20 sec and centrifuged again for 10 min at 3000 x rpm. After centrifugation, the top phase was collected in a cuvette and the absorbance at 520 nm (Abs_{520}) was measured. The absorbance values were normalized calculating the ratio Abs_{520}/OD_{600}.

Congo red binding assay

Congo red binding assay was performed as described previously (14). Briefly, 40 µg/ml of Congo Red and 15 µg/ml of Coomassie brilliant blue were added to 1.5%-agar TB plates, and 2 µl of bacterial cultures at OD_{600} of 0.04 were spotted. Plates were incubated for 24 hrs at 30°C, followed by 48 hrs at room temperature. All strains were tested at least three times.

Exotoxin A quantification

Quantification of exotoxin A, either intracellular or secreted in culture medium supernatants, was performed as follows. *P. aeruginosa* cells grown in LB medium in LSha or LSta conditions were diluted to a final OD_{600} of 3.0 using a fresh LB medium. *P. aeruginosa* cells grown in CBio were directly resuspended in PBS to a final OD_{600} equal to 3.0. 7.5 ml of cell suspension was centrifuged, and supernatants were separated from pelleted cells. 100 µl of supernatants added to 100 µl of 2× LDS Sample Buffer (GenScript). Pelleted cells were resuspended in PBS at OD_{600} of 3 and lysed using a Constant Systems CF1 Cell Disrupter at 28,000 psi. The sample protein content was assessed by a Biospectrometer (Eppendorf). 30 µl of supernatant preparation and 60 µg of total proteins deriving from pelleted cells were denatured at 95°C in LDS Sample Buffer (GenScript) and loaded onto a 4-12% polyacrylamide gel (GenScript). Electrophoresis (PAGE) was performed at 120 V in MES-SDS running buffer. Proteins resolved by SDS-PAGE were transferred onto Amersham Protran 0.45 µm NC Nitrocellulose blotting membrane using a semi-dry electro-blotting apparatus (Fastblot B33, Biometra) set at 56 mA, 300 V for 1 hour. The blot membranes were successively probed for exotoxin A using goat polyclonal anti-exotoxin A antibodies

(LifeSpan Biosciences; Seattle, WA, USA) and rabbit anti-goat IgG FITC-conjugated secondary antibody (LifeSpan Biosciences; Seattle, WA, USA). Bands were visualized by the filter Alexa488 with a ChemiDoc™ MP imaging system, using the ImageLab analysis software. (Bio-Rad; Hercules, CA, USA).

References

1. Abril MA, Michan C, Timmis KN, Ramos JL. 1989. Regulator and enzyme specificities of the TOL plasmid-encoded upper pathway for degradation of aromatic hydrocarbons and expansion of the substrate range of the pathway. J Bacteriol 171:6782–90.
2. Ferrara S, Carloni S, Fulco R, Falcone M, Macchi R, Bertoni G. 2015. Post-transcriptional regulation of the virulence-associated enzyme AlgC by the σ 22 -dependent small RNA ErsA of Pseudomonas aeruginosa. Environ Microbiol 17:199–214.
3. Delvillani F, Sciandrone B, Peano C, Petiti L, Berens C, Georgi C, Ferrara S, Bertoni G, Pasini ME, Dehò G, Briani F. 2014. Tet-Trap, a genetic approach to the identification of bacterial RNA thermometers: application to Pseudomonas aeruginosa. RNA 20:1963–1976.
4. Martínez-García E, de Lorenzo V. 2011. Engineering multiple genomic deletions in Gram-negative bacteria: Analysis of the multi-resistant antibiotic profile of Pseudomonas putida KT2440. Environ Microbiol 13:2702–2716.
5. Corcoran CP, Podkaminski D, Papenfort K, Urban JH, Hinton JCD, Vogel J. 2012. Superfolder GFP reporters validate diverse new mRNA targets of the classic porin regulator, MicF RNA. Mol Microbiol 84:428–445.
6. Ferrara S, Brugnoli M, De Bonis A, Righetti F, Delvillani F, Dehò G, Horner D, Briani F, Bertoni G. 2012. Comparative Profiling of Pseudomonas aeruginosa Strains Reveals Differential Expression of Novel Unique and Conserved Small RNAs. PLoS One 7:e36553.
7. Ferrara S, Carrubba R, Santoro S, Bertoni G. 2021. The Small RNA ErsA Impacts the Anaerobic Metabolism of Pseudomonas aeruginosa Through Post-Transcriptional Modulation of the Master Regulator Anr. Front Microbiol 12:1–14.

8. Carty NL, Rumbaugh KP, Hamood AN. 2003. Regulation of toxA by PtxR in Pseudomonas aeruginosa PA103. Can J Microbiol 49:450–464.
9. Livak KJ, Schmittgen TD. 2001. Analysis of relative gene expression data using real-time quantitative PCR and the 2(-Delta Delta C(T)) Method. Methods 25:402–408.
10. Dobin A, Davis CA, Schlesinger F, Drenkow J, Zaleski C, Jha S, Batut P, Chaisson M, Gingeras TR. 2013. STAR: Ultrafast universal RNA-seq aligner. Bioinformatics 29:15–21.
11. Li H, Handsaker B, Wysoker A, Fennell T, Ruan J, Homer N, Marth G, Abecasis G, Durbin R. 2009. The Sequence Alignment/Map format and SAMtools. Bioinformatics 25:2078–2079.
12. Love MI, Huber W, Anders S. 2014. Moderated estimation of fold change and dispersion for RNA-seq data with DESeq2. Genome Biol 15:1–21.
13. Essar DW, Eberly L, Hadero A, Crawford IP. 1990. Identification and characterization of genes for a second anthranilate synthase in Pseudomonas aeruginosa: interchangeability of the two anthranilate synthases and evolutionary implications. J Bacteriol 172:884–900.
14. Römling U, Bian Z, Hammar M, Sierralta WD, Normark S. 1998. Curli fibers are highly conserved between Salmonella typhimurium and Escherichia coli with respect to operon structure and regulation. J Bacteriol 180:722–731.
