# Supplementary material for: Multifaceted Interplay between Hfq and the Small RNA GssA in Pseudomonas aeruginosa
Source: mBio. 2022 Dec 8;14(1):e02418-22. doi: 10.1128/mbio.02418-22 (PMC9973299; doi:10.1128/mbio.02418-22)
Supplement: TABLE S2 [file mbio.02418-22-st002.pdf]

TAB S2A Differentially expressed genes (DEGs) in PA14Δ*gssA* compared with PA14.

Up-regulated genes in the Δ*gssA* mutant

Gene ID	Gene description	Fold Change	log_2Fold Change	p value	Adj *p* value
	**Glucose transport and metabolism**				
PA14_22890	*gapA*, glyceraldehyde-3-phosphate dehydrogenase	4.80	2.26	4.57E-24	2.76E-21
PA14_22910	*edd*, phosphogluconate dehydratase	4.26	2.09	1.05E-28	1.91E-25
PA14_22930	*glk*, glucokinase	4.37	2.13	2.98E-17	9.01E-15
PA14_22940	*gltR*, two-component response regulator GltR	4.19	2.07	1.80E-21	8.91E-19
PA14_22960	*gtrS*, two-component sensor GtrS	3.26	1.71	1.83E-09	3.11E-07
PA14_23060	*hexR*, DNA-binding transcriptional regulator HexR	2.05	1.03	2.58E-06	2.15E-04
PA14_23070	*zwf*, glucose-6-phosphate 1-dehydrogenase	5.44	2.44	3.62E-31	9.84E-28
PA14_23080	*pgl*, 6-phosphogluconolactonase	10.01	3.32	5.99E-24	3.26E-21
PA14_23090	keto-hydroxyglutarate-aldolase/ keto-deoxy-phosphogluconate aldolase	9.73	3.28	3.69E-40	2.00E-36
PA14_34600	glyceraldehyde-3-phosphate dehydrogenase	1.74	0.80	2.83E-05	1.69E-03
PA14_34610	hypothetical protein	14.30	3.84	9.51E-09	1.26E-06
PA14_34630	*gnuT*, gluconate permease (GntP in PAO1)	17.68	4.14	1.49E-28	2.02E-25
PA14_34640	gluconokinase	4.72	2.24	9.21E-14	2.50E-11
PA14_34660	*gntR*, transcriptional regulator GntR	3.89	1.96	1.14E-18	4.12E-16
PA14_35290	*gnd*, gluconate dehydrogenase	1.86	0.89	3.18E-06	2.47E-04
PA14_35300	hypothetical protein	2.05	1.03	5.56E-07	5.49E-05
PA14_35320	2-hydroxyacid dehydrogenase	2.86	1.52	2.03E-12	5.00E-10
PA14_35330	2-ketogluconate transporter (KguT in PAO1)	2.13	1.09	3.72E-07	4.04E-05
PA14_35340	2-ketogluconate kinase	2.26	1.18	2.96E-05	1.75E-03
PA14_35360	hypothetical protein	2.13	1.09	5.19E-05	2.91E-03
PA14_35370	*ptxS*, transcriptional regulator PtxS	1.65	0.72	2.74E-03	6.89E-02
	**Type VI Secretion System (T6SS)**				
PA14_00820	*tagQ1*, HCP secretion island I (H1-T6SS)	1.77	0.83	5.30E-05	2.94E-03
PA14_00875	*ppkA*, HCP secretion island I (H1-T6SS)	1.55	0.63	4.42E-03	9.99E-02
PA14_00890	*pppA*, HCP secretion island I (H1-T6SS)	1.98	0.99	8.15E-05	4.10E-03
PA14_01040	*tagJ1*, HCP secretion island I (H1-T6SS)	1.82	0.87	1.58E-03	4.76E-02
PA14_01060	*tssE1*, HCP secretion island I (H1-T6SS)	2.34	1.23	6.29E-05	3.28E-03
PA14_01070	*tssF1*, HCP secretion island I (H1-T6SS)	2.35	1.24	5.68E-07	5.51E-05
PA14_01080	*tssG1*, HCP secretion island I (H1-T6SS)	1.99	0.99	3.26E-06	2.50E-04
PA14_01110	*vgrG1a*, HCP secretion island I (H1-T6SS)	2.00	1.00	1.59E-05	1.04E-03
PA14_01200	type VI effector protein	1.44	0.53	3.76E-03	8.96E-02
PA14_42950	*fha2*, HCP secretion island II (H2-T6SS)	1.53	0.61	3.34E-03	8.13E-02
	**Respiratory chain**				
PA14_01320	coIII .cytochrome c oxidase subunit III	1.67	0.74	3.96E-03	9.28E-02
PA14_06650	*nirN*, c-type cytochrome	2.55	1.35	3.69E-05	2.13E-03
PA14_06660	*nirE*, uroporphyrin-III c-methyltransferase	2.41	1.27	1.79E-03	5.29E-02
PA14_06670	*nirJ*, heme d1 biosynthesis protein NirJ	2.54	1.34	1.76E-04	7.67E-03
PA14_06680	*nirH*, hypothetical protein	2.27	1.18	2.26E-03	6.08E-02

PA14_06690	*nirG*, transcriptional regulator	2.60	1.38	2.05E-03	5.84E-02
PA14_06810	*norC*, nitric-oxide reductase subunit C	3.20	1.68	5.73E-05	3.08E-03
	**Fatty acids biosynthesis and metabolism**				
PA14_20940	acyl carrier protein	1.69	0.76	2.16E-03	6.01E-02
PA14_20950	fabH2. 3-oxoacyl-ACP synthase	2.16	1.11	8.42E-09	1.14E-06
PA14_20960	isomerase	1.99	0.99	9.18E-05	4.37E-03
PA14_20970	cyp23. cytochrome P450	2.04	1.03	1.95E-05	1.19E-03
PA14_20980	short chain dehydrogenase	1.86	0.89	1.68E-04	7.52E-03
PA14_21000	hypothetical protein	2.23	1.16	8.42E-05	4.16E-03
PA14_21010	FAD-dependent monooxygenase	2.12	1.08	4.98E-07	5.10E-05
PA14_21020	non-ribosomal peptide synthetase	1.72	0.78	1.21E-03	3.89E-02
PA14_41170	*fabI*. NADH-dependent enoyl-ACP reductase	1.47	0.56	3.15E-03	7.70E-02
PA14_52800	acsA ,acetyl-CoA synthetase	1.53	0.61	1.20E-03	3.89E-02
	**Pyochelin biosynthesis**				
PA14_09210	*pchA*, salicylate biosynthesis isochorismate synthase	2.18	1.12	1.36E-04	6.24E-03
PA14_09230	*pchC*, pyochelin biosynthetic protein PchC	2.55	1.35	3.93E-03	9.23E-02
PA14_09270	*pchE*, dihydroaeruginoic acid synthetase	2.22	1.15	3.89E-03	9.23E-02
PA14_09280	*pchF*, pyochelin synthetase	2.39	1.26	3.19E-06	2.47E-04
PA14_09350	*fptB*, possible role in transport of Fe-pyochelin	1.99	0.99	6.64E-04	2.51E-02
	**Hemolysis/hemagglutination**				
PA14_00490	hemolysin activation/secretion protein	1.79	0.84	1.02E-03	3.44E-02
PA14_00510	hemagglutinin	1.63	0.70	4.96E-04	1.92E-02
	**Oxidative stress**				
PA14_30280	*trxB1*, thioredoxin reductase 1	1.48	0.57	2.84E-03	7.07E-02
	**General secretion system**				
PA14_67720	*secB*, preprotein translocase subunit SecB	1.78	0.83	5.45E-04	2.07E-02
	**Biosynthesis of secondary metabolites**				
PA14_06920	class III pyridoxal phosphate-dependent aminotransferase	2.37	1.24	1.04E-05	7.08E-04
PA14_06930	glutamine amidotransferase	2.26	1.18	3.54E-05	2.07E-03
	**Outer membrane porins**				
PA14_02020	outer membrane porin	1.65	0.73	1.88E-03	5.50E-02
PA14_03800	*oprE*, outer membrane porin OprE precursor	1.54	0.63	3.23E-04	1.29E-02
	**Fructose and mannose metabolism**				
PA14_18260	*fruK*, 1-phosphofructokinase	1.61	0.68	8.04E-04	2.89E-02
	**Oxidative phosphorylation**				
PA14_11690	*ppa*, inorganic pyrophosphatase	1.60	0.68	4.13E-03	9.50E-02

Down-regulated genes in the Δ*gssA* mutant

Gene ID	Gene description	Fold Change	log_2Fold Change	p value	Adj *p* value
	**Respiratory chain**				
PA14_29850	*nuoN*, NADH dehydrogenase subunit N	-4.14	-2.05	2.48E-25	2.25E-22
PA14_29860	*nuoM*, NADH dehydrogenase subunit M	-4.36	-2.12	4.21E-21	1.90E-18
PA14_29880	*nuoL*, NADH dehydrogenase subunit L	-4.10	-2.03	3.20E-28	3.47E-25
PA14_29890	*nuoK*, NADH dehydrogenase subunit K	-3.80	-1.92	4.01E-12	9.07E-10
PA14_29900	*nuoJ*, NADH dehydrogenase subunit J	-3.40	-1.77	1.16E-09	2.04E-07
PA14_29920	*nuoI*, NADH dehydrogenase subunit I	-2.93	-1.55	1.10E-06	9.48E-05
PA14_29930	*nuoH*, NADH dehydrogenase subunit H	-3.59	-1.84	6.85E-17	1.96E-14
PA14_29940	*nuoG*, NADH dehydrogenase subunit G	-3.70	-1.89	1.44E-17	4.59E-15
PA14_29970	*nuoF*, NADH dehydrogenase I subunit F	-4.38	-2.13	6.57E-18	2.23E-15
PA14_29980	*nuoE*, NADH dehydrogenase subunit E	-3.66	-1.87	8.76E-25	6.80E-22
PA14_29990	*nuoD*, bifunctional NADH:ubiquinone oxidoreductase subunit C/D	-3.89	-1.96	3.12E-13	8.06E-11
PA14_30010	*nuoB*, NADH dehydrogenase subunit B	-3.81	-1.93	1.19E-19	4.61E-17
PA14_30020	*nuoA*, NADH dehydrogenase subunit A	-4.59	-2.20	1.00E-24	6.81E-22
PA14_24860	*snr1*, cytochrome c Snr1	-1.79	-0.84	8.89E-05	4.28E-03
PA14_13030	*cioA*, CioA, cyanide insensitive terminal oxidase	-2.12	-1.08	5.78E-09	8.06E-07
PA14_13040	*cioB*, CioB, cyanide insensitive terminal oxidase	-1.89	-0.92	1.67E-05	1.06E-03
	**Type III secretion system (T3SS)**				
PA14_00560	*exoT*, exoenzyme T (T3SS effector)	-1.56	-0.64	7.24E-04	2.67E-02
PA14_36345	*exoY*, adenylate cyclase (T3SS effector)	-1.66	-0.73	2.78E-05	1.68E-03
PA14_42440	*popD*, translocator outer membrane protein PopD precursor (T3SS)	-1.65	-0.73	3.04E-04	1.22E-02
PA14_42450	*popB*, translocator protein PopB (T3SS)	-1.83	-0.87	1.86E-05	1.15E-03
PA14_42460	*pcrH*, regulatory protein PcrH (T3SS)	-1.74	-0.80	3.99E-04	1.57E-02
PA14_42470	*pcrV*, secretion protein PcrV (T3SS)	-1.81	-0.86	7.25E-05	3.68E-03
PA14_25180	*psrA*, transcriptional regulator PsrA (T3SS regulation)	-1.85	-0.89	8.35E-05	4.16E-03
	**Adhesion to epithelial cells**				
PA14_31290	*lecA*, PA-I galactophilic lectin	-1.92	-0.94	4.31E-06	3.21E-04
	**Membrane transport**				
PA14_01580	*gabP*, gamma-aminobutyrate permease	-2.72	-1.44	9.70E-07	8.68E-05
PA14_09540	*mexG*, transmembrane transport	-2.11	-1.08	1.84E-03	5.39E-02
PA14_28170	formate/nitrate transporter	-1.84	-0.88	1.82E-04	7.86E-03
PA14_38560	MFS transporter	-2.29	-1.20	1.25E-03	3.99E-02
PA14_40240	ABC transporter ATP-binding protein/permease	-3.37	-1.75	2.75E-09	4.27E-07
	**Valine, leucine and isoleucine degradation**				
PA14_18120	*mmsA*, methylmalonate-semialdehyde dehydrogenase	-2.70	-1.43	1.08E-06	9.46E-05
PA14_18140	*mmsB*, 3-hydroxyisobutyrate dehydrogenase	-2.50	-1.32	3.19E-06	2.47E-04
	**Protease activity**				
PA14_04650	*pfpI*, PfpI protease	-1.49	-0.58	2.15E-03	6.01E-02
PA14_09900	*prpL*, PvdS-regulated endoprotease	-2.02	-1.01	1.79E-05	1.12E-03
PA14_40290	lasA, LasA protease	-1.95	-0.96	1.55E-03	4.71E-02
	**Hydrolysis of short-chain aliphatic amides**				
PA14_20560	*amiE*, acylamide amidohydrolase	-1.63	-0.71	6.71E-04	2.51E-02

	**Transcription regulation**				
PA14_38500	*hmgR*, transcriptional repressor HmgR	-1.63	-0.70	3.06E-03	7.53E-02
PA14_45960	*lasR*, transcriptional regulator LasR	-1.45	-0.53	4.39E-03	9.98E-02
	**Stress response**				
PA14_36810	*katE*, hydroperoxidase II (response to oxidative stress)	-2.16	-1.11	4.10E-04	1.60E-02
PA14_36820	Stress-induced hypothetical protein	-3.31	-1.73	2.88E-07	3.19E-05
PA14_64480	*osmE*, osmotically inducible lipoprotein	-1.75	-0.80	4.80E-05	2.71E-03
	**Trehalose metabolism**				
PA14_33450	*treA*, trehalase	-2.25	-1.17	1.34E-03	4.23E-02
PA14_33480	*sndH*, L-sorbosone dehydrogenase	-2.15	-1.11	2.63E-03	6.70E-02
PA14_36580	glycosyl hydrolase	-2.85	-1.51	7.21E-05	3.68E-03
PA14_36605	maltooligosyl trehalose synthase	-2.82	-1.50	8.76E-05	4.28E-03
PA14_36730	trehalose synthase	-2.25	-1.17	2.44E-03	6.39E-02
	**Glycogen transport and metabolism**				
PA14_36570	*glgA*, glycogen synthase	-3.08	-1.62	3.62E-08	4.57E-06
PA14_36590	4-alpha-glucanotransferase	-2.35	-1.23	2.48E-03	6.47E-02
PA14_36630	glycosyl hydrolase	-2.51	-1.33	2.84E-06	2.34E-04
PA14_36710	*glgB*, glycogen branching protein	-2.28	-1.19	7.25E-05	3.68E-03
PA14_36740	*glgE*	-2.53	-1.34	8.89E-05	4.28E-03
PA14_36840	*glgP*, glycogen phosphorylase	-2.51	-1.33	6.21E-05	3.28E-03
	**Type II secretion system**				
PA14_55890	type II secretion system protein	-2.68	-1.42	2.12E-09	3.39E-07
PA14_55920	type II secretion system protein	-2.49	-1.32	9.75E-07	8.68E-05
	**Type IV pilus assembly**				
PA14_55790	Putative pilus assembly protein	-2.51	-1.33	7.87E-10	1.47E-07
PA14_55820	*tadG*	-2.29	-1.20	2.43E-07	2.75E-05
PA14_55850	*tadD*	-2.38	-1.25	1.60E-05	1.04E-03
PA14_55860	*tadC*	-2.71	-1.44	2.29E-07	2.64E-05
PA14_55880	*tadB*	-2.92	-1.54	7.39E-10	1.43E-07
PA14_55900	*tadZ*	-2.67	-1.42	5.17E-07	5.20E-05
PA14_55930	*rcpC*	-2.62	-1.39	4.66E-07	4.87E-05
PA14_55940	*flp*	-5.75	-2.52	3.60E-20	1.50E-17
	**Chaperon-Usher fimbriae assembly**				
PA14_61500	*cupE1*	-2.29	-1.20	5.95E-05	3.17E-03
PA14_61510	*cupE2*	-2.60	-1.38	2.36E-03	6.29E-02
PA14_61540	*cupE5*	-1.73	-0.79	2.96E-03	7.35E-02
	**Chemotaxis**				
PA14_55960	*pctC*, chemotactic transducer PctC	-1.52	-0.60	1.45E-03	4.48E-02
	**Fatty acids metabolism**				
PA14_13110	long-chain-fatty-acid-CoA ligase	-1.64	-0.71	3.01E-04	1.22E-02
PA14_27730	*fadE*, acyl-CoA dehydrogenase	-1.59	-0.67	1.61E-03	4.83E-02
PA14_54660	enoyl-CoA hydratase/isomerase	-1.53	-0.62	1.11E-03	3.71E-02
	**Glycerophospholipid metabolism**				
PA14_36690	cardiolipin synthase 2	-2.38	-1.25	1.50E-03	4.59E-02
	**Amino acids metabolism**				

PA14_01610	*bauB*	-2.79	-1.48	4.82E-08	5.82E-06
PA14_01620	*aptA*, beta alanine-pyruvate transaminase	-3.82	-1.93	2.06E-09	3.39E-07
PA14_19370	asparagine synthetase	-1.62	-0.69	3.76E-04	1.49E-02
PA14_36360	hypothetical protein	-3.54	-1.83	5.94E-06	4.25E-04
PA14_36370	carboxylate-amine ligase	-2.09	-1.06	2.15E-03	6.01E-02
PA14_38530	*fahA*, fumarylacetoacetase	-2.02	-1.01	2.04E-03	5.83E-02
PA14_38550	*maiA*, maleylacetoacetate isomerase	-2.23	-1.16	1.38E-03	4.35E-02

TAB S2B Differential expressed genes (DEGs) in PA14Δ*gssA* strain compared with PA14 annotated as coding hypothetical proteins or sharing similarity with characterized proteins.

Up-regulated genes in the Δ*gssA* mutant

Gene ID	Gene description	Fold Change	log_2Fold Change	p value	Adj *p* value
PA14_02530	hypothetical protein	1.95	0.96	9.77E-04	3.36E-02
PA14_06710	transcriptional regulator	2.51	1.33	9.62E-04	3.33E-02
PA14_06890	hypothetical protein	2.22	1.15	8.02E-07	7.51E-05
PA14_06900	hypothetical protein	3.06	1.61	1.53E-05	1.02E-03
PA14_09380	transporter	2.07	1.05	5.04E-04	1.94E-02
PA14_14360	sodium:sulfate symporter	1.48	0.57	2.24E-03	6.04E-02
PA14_16190	hypothetical protein	1.58	0.66	3.76E-03	8.96E-02
PA14_19690	hypothetical protein	1.76	0.81	4.36E-03	9.96E-02
PA14_20900	MFS transporter	1.93	0.95	9.99E-05	4.68E-03
PA14_20920	hypothetical protein	1.97	0.98	4.29E-06	3.21E-04
PA14_21700	two-component sensor	1.41	0.49	2.73E-03	6.89E-02
PA14_31130	hypothetical protein	2.73	1.45	5.40E-05	2.96E-03
PA14_31150	hypothetical protein	2.40	1.26	5.79E-12	1.26E-09
PA14_32780	hypothetical protein	1.77	0.82	1.63E-04	7.38E-03
PA14_35270	cytochrome c precursor	1.85	0.89	1.22E-05	8.21E-04
PA14_43160	transporter	1.73	0.79	1.25E-03	3.99E-02
PA14_46080	hypothetical protein	10.52	3.39	3.59E-09	5.42E-07
PA14_46100	transporter	3.57	1.83	1.88E-04	7.99E-03
PA14_46140	hypothetical protein	3.62	1.86	9.99E-04	3.39E-02
PA14_60570	hypothetical protein	1.69	0.76	2.62E-03	6.70E-02
PA14_64840	short-chain dehydrogenase	2.04	1.03	9.18E-04	3.20E-02

Down-regulated genes in the Δ*gssA* mutant

Gene ID	Gene description	Fold Change	log_2Fold Change	p value	Adj *p* value
PA14_00310	peptidyl-prolyl isomerase	-1.53	-0.61	2.24E-03	6.04E-02
PA14_00320	hypothetical protein	-1.78	-0.83	1.39E-03	4.35E-02
PA14_00480	hypothetical protein	-1.43	-0.51	3.38E-03	8.17E-02
PA14_01600	aldehyde dehydrogenase	-3.21	-1.68	3.95E-08	4.87E-06
PA14_07370	hypothetical protein	-1.98	-0.99	3.09E-06	2.47E-04
PA14_09930	exonuclease III	-2.50	-1.32	4.43E-03	9.99E-02
PA14_10550	sulfite or nitrite reductase	-1.44	-0.53	3.72E-03	8.95E-02
PA14_13630	hypothetical protein	-1.81	-0.86	2.79E-03	6.97E-02
PA14_15120	hypothetical protein	-1.91	-0.93	1.16E-03	3.81E-02
PA14_15130	hypothetical protein	-1.81	-0.85	2.70E-03	6.85E-02
PA14_15770	hypothetical protein	-1.52	-0.60	2.60E-03	6.69E-02
PA14_18800	hypothetical protein	-1.65	-0.73	2.60E-04	1.08E-02

PA14_20460	hypothetical protein	-1.78	-0.83	2.62E-04	1.08E-02
PA14_20570	chaperone	-1.50	-0.59	3.06E-03	7.53E-02
PA14_21670	hypothetical protein	-2.65	-1.41	4.19E-07	4.47E-05
PA14_21680	hypothetical protein	-1.70	-0.76	1.65E-03	4.90E-02
PA14_22400	hypothetical protein	-2.67	-1.42	3.60E-08	4.57E-06
PA14_23100	hypothetical protein	-1.95	-0.97	5.30E-06	3.84E-04
PA14_24630	hypothetical protein	-1.84	-0.88	1.62E-03	4.85E-02
PA14_24760	hypothetical protein	-1.47	-0.55	1.07E-03	3.58E-02
PA14_26050	transporter	-1.76	-0.82	4.24E-03	9.73E-02
PA14_26780	hypothetical protein	-1.68	-0.74	4.79E-05	2.71E-03
PA14_28600	hypothetical protein	-1.59	-0.67	1.70E-04	7.52E-03
PA14_31160	hypothetical protein	-3.19	-1.68	2.83E-12	6.69E-10
PA14_31170	hypothetical protein	-3.92	-1.97	9.70E-10	1.76E-07
PA14_31350	hypothetical protein	-1.52	-0.60	9.14E-04	3.20E-02
PA14_31360	hypothetical protein	-1.64	-0.71	1.20E-03	3.89E-02
PA14_33160	hypothetical protein	-2.04	-1.03	1.70E-04	7.52E-03
PA14_33460	hypothetical protein	-2.08	-1.06	2.32E-04	9.70E-03
PA14_33870	hypothetical protein	-1.63	-0.70	4.05E-03	9.37E-02
PA14_35570	pslN, hypothetical protein	-1.78	-0.83	1.45E-03	4.48E-02
PA14_36375	hypothetical protein	-2.17	-1.12	1.62E-04	7.37E-03
PA14_36390	hypothetical protein	-2.43	-1.28	1.14E-03	3.76E-02
PA14_36410	hypothetical protein	-3.07	-1.62	6.15E-06	4.34E-04
PA14_36450	hypothetical protein	-1.78	-0.83	2.58E-03	6.67E-02
PA14_36460	hypothetical protein	-1.93	-0.95	9.30E-05	4.40E-03
PA14_36480	hypothetical protein	-2.15	-1.10	8.65E-04	3.07E-02
PA14_36500	hypothetical protein	-2.50	-1.32	1.30E-04	6.02E-03
PA14_36520	hypothetical protein	-2.76	-1.46	9.45E-07	8.68E-05
PA14_36530	hypothetical protein	-2.74	-1.45	8.32E-06	5.72E-04
PA14_36550	hypothetical protein	-2.27	-1.19	2.68E-04	1.10E-02
PA14_36560	hypothetical protein	-2.38	-1.25	2.08E-07	2.45E-05
PA14_36650	hypothetical protein	-2.59	-1.37	5.04E-06	3.70E-04
PA14_36660	alcohol dehydrogenase	-2.20	-1.14	2.22E-03	6.04E-02
PA14_36670	hypothetical protein	-2.29	-1.20	1.98E-03	5.74E-02
PA14_36680	hypothetical protein	-2.55	-1.35	9.97E-04	3.39E-02
PA14_36700	hypothetical protein	-2.82	-1.50	1.73E-05	1.09E-03
PA14_36760	KU domain-containing protein	-2.02	-1.01	7.77E-04	2.83E-02
PA14_36780	hypothetical protein	-2.58	-1.36	5.36E-04	2.05E-02
PA14_36850	hypothetical protein	-2.35	-1.24	2.04E-03	5.83E-02
PA14_36870	short-chain dehydrogenase	-2.51	-1.33	2.44E-06	2.07E-04
PA14_36960	transporter	-2.52	-1.33	3.38E-03	8.17E-02
PA14_36980	hypothetical protein	-2.33	-1.22	1.76E-04	7.67E-03
PA14_38370	hypothetical protein	-1.76	-0.82	2.28E-03	6.09E-02
PA14_39520	hydroxylase large subunit	-1.98	-0.98	2.50E-03	6.51E-02
PA14_39540	ferredoxin	-1.60	-0.68	2.11E-03	5.97E-02
PA14_39780	hypothetical protein	-3.40	-1.76	6.53E-07	6.22E-05

PA14_40230	secretion protein	-3.21	-1.68	9.44E-12	1.97E-09
PA14_40250	outer membrane protein	-2.85	-1.51	4.98E-09	7.31E-07
PA14_40260	hypothetical protein	-3.34	-1.74	2.36E-10	4.75E-08
PA14_40300	hypothetical protein	-1.71	-0.78	4.01E-03	9.30E-02
PA14_40750	hypothetical protein	-1.51	-0.60	2.42E-03	6.39E-02
PA14_44230	hypothetical protein	-2.21	-1.14	2.04E-03	5.83E-02
PA14_44260	oxidoreductase	-1.89	-0.91	2.17E-03	6.01E-02
PA14_46280	hypothetical protein	-2.11	-1.08	2.30E-04	9.69E-03
PA14_49910	hypothetical protein	-1.41	-0.49	3.98E-03	9.29E-02
PA14_51940	hypothetical protein	-1.85	-0.89	7.92E-04	2.87E-02
PA14_54750	hypothetical protein	-1.95	-0.97	8.38E-04	3.00E-02
PA14_55110	hypothetical protein	-1.63	-0.71	1.21E-03	3.89E-02
PA14_55780	two-component sensor	-1.81	-0.86	2.20E-03	6.03E-02
PA14_56030	hypothetical protein	-1.42	-0.51	2.20E-03	6.03E-02
PA14_56040	hypothetical protein	-1.54	-0.62	9.16E-04	3.20E-02
PA14_56050	hypothetical protein	-1.60	-0.68	1.50E-03	4.59E-02
PA14_56910	hypothetical protein	-1.49	-0.57	3.92E-03	9.23E-02
PA14_60950	hypothetical protein	-1.40	-0.48	2.39E-03	6.33E-02
PA14_62380	hypothetical protein	-1.70	-0.76	7.27E-04	2.67E-02
PA14_62680	hypothetical protein	-1.91	-0.93	1.84E-04	7.86E-03
PA14_62690	hypothetical protein	-1.71	-0.78	7.21E-04	2.67E-02
PA14_72360	hypothetical protein	-1.72	-0.79	5.51E-05	2.99E-03
PA14_72370	hypothetical protein	-2.01	-1.01	5.32E-09	7.61E-07
