# Supplementary material for: Multifaceted Interplay between Hfq and the Small RNA GssA in Pseudomonas aeruginosa
Source: mBio. 2022 Dec 8;14(1):e02418-22. doi: 10.1128/mbio.02418-22 (PMC9973299; doi:10.1128/mbio.02418-22)
Supplement: TABLE S3 [file mbio.02418-22-st003.pdf]

**TAB S3A** Oligonucleotides

| Oligo # | Oligo Name | Sequence (5' → 3')[a] |
|---|---|---|
| 1 | SPA0012 | CAATTACCGGCGCGGTAGG |
| 2 | 5'BIOTIN-SPA0012 | [BIO]CAATTACCGGCGCGGTAGG |
| 3 | PA5SRNA01 | GAGCTTGACGATGACCTACTCTCACATG |
| 4 | 5'BIOTIN-PA5SRNA01 | [BIO]GAGCTTGACGATGACCTACTCTCACATG |
| 5 | PE_SPA0012_rev | GCCGCTGATAGCCTTTACAC |
| 6 | +601_for | ACCTACCGCGAGGAAGAAAT |
| 7 | +1683_rev | TTCGATACCCTTCTGGATCG |
| 8 | Primer_#32r | GGTGGTGACGTCCTTTCATG |
| 9 | NcoI_5'SPA0012_for | GAccatggGCAGCTACCACCCAAGG |
| 10 | PstI_3'SPA0012_rev | GAActgcagGGTAGCGAAGAAGGTCTAGAA |
| 11 | PstI_3'SPA0012_GUGhfqMUT_rev | GAActgcagGGTAGCGAAGAAGGTCTAGAAAAAGAACACCAGCTACC |
| 12 | TS1_gssA_SacI_for | CATgagctcAAATCCTTGGCAATGTTCCG |
| 13 | TS1_gssA_rev | GTCTAGAAAAAGAAGTGCGCGGCCGCGATCAGCCCTCCTGCAATT |
| 14 | TS2_gssA_for | GCGGCCGCGCACTTCTTTTTCTAGAC |
| 15 | TS2_gssA_BamHI_rev | CGggatccGTAGCCGTTGCGCAGCGAAA |
| 16 | gssA_300_up_for | TCAGAACGCTAAGTGAGGG |
| 17 | gssA_300_down_rev | CGAGATCAGCCTGAAGAACAG |
| 18 | -464ATG_hfq_Nsi_for | GTTTTatgcatAGCTGGCGATTGCTCCCG |
| 19 | +108ATG_ hfq_NheI_rev | GTTTTgctagcGATCTGGCCTTGCAGCTTG |
| 20 | PtetO1_ClaI_for_nw | GTTTTatcgatCGTCTTCACCTCGAGTC |
| 21 | sfGFP_TAA_XbaI_rev | TGATGCCtctagaTTATTTGTAGAGCTC |
| 22 | sfGFP_+96_rev | TTGTGCCCATTAACATCACCATC |
| 23 | pHERD_for | ATCGCAACTCTCTACTGTTTCT |
| 24 | pHERD_rev | CAGCTATGACCATGATTACGCC |
| 25 | pSEVA_for | TAAAACGACGGCCAGTATAGGGATA |
| 26 | pSEVA_rev | CAGCTATGACCATGATTACGCC |
| 27 | M13_for | GTAAAACGACGGCCAG |
| 28 | M13_rev | CAGGAAACAGCTATGACC |
| 29 | q16S_for | TGTCGTCAGCTCGTGTCGTGA |
| 30 | q16S_rev | ATCCCCACCTTCCTCCGGT |
| 31 | qSpa12_for | CGTGGGGTGTAAAGGCTATCA |
| 32 | qSpa12_rev | TTTCCCATTGCCCAAACCGG |
| 33 | qPtxS_for | CGCGTCACCATCAACCAG |
| 34 | qPtxS_rev | CATCTGGTTGGGGCGGTA |
| 35 | qPtxR_for | CTTCGTCAAGTGGCACATCAGC |
| 36 | qPtxR_rev | GGGAAACTGCCGGATGAACG |
| 37 | qToxA_for_B | CTCAAGCTGGCCATCGACAAC |
| 38 | qToxA_rev | GGTTACCGGCGTTCAGTTCGT |
| 39 | qToxR(RegA)_for | ACTGCGACAGACAGAACGCC |

| | | |
|---|---|---|
| 40 | qToxR(RegA)_rev | CGAATCCGTCGTTCGCATCAC |
| 41 | qPvcB_for | AGAAATTCCCCATCCTGCGTTTC |
| 42 | qPvcB_rev | GTCGTAGTGGAACTCGGAGG |
| 43 | qHfq_for | AATACCCTGCGGAAAGAACG |
| 44 | qHfq_rev | CGCGTGCTTGTAAACCATCT |
| 45 | qCrcZ for | CAACACGTAACGACTCCAGC |
| 46 | qCrcZ rev | GAGCGATCCAACCAGTCTGT |

[a] [BIO]: Biotin. Lowercase: sites for restriction enzymes.

**TAB S3B** Strains and Plasmids

| **Strains or plasmids** | **Genotype or description** | **Reference** |
|---|---|---|
| *Pseudomonas aeruginosa* | | |
| PA14 | wild-type | (1) |
| PA14 Δ*gssA* | markerless Δ*gssA* | this work |
| PA14 Δ*hfq* | markerless Δ*hfq* | (2) |
| PA14 Δ*hfq*Δ*gssA* | markerless Δ*hfq*Δ*gssA* | this work |
| PA14 Δ *rpoS* | Δ*rpoS*, $Gm^r$ | (3) |
| PA14 Δ *rpoN* | Δ*rpoN*, $Gm^r$ | (3) |
| PA14 Δ *pvdS* | Δ*pvdS*, $Gm^r$ | (3) |
| PA14 Δ *sigX* | Δ*sigX*, $Gm^r$ | (3) |
| PA14 Δ *fpvI* | Δ*fpvI*, $Gm^r$ | (3) |
| PA14 Δ*algU* | markerless Δ*algU* | (4) |
| *Escherichia coli* | | |
| TOP10 | *mcrA* Δ( *mrr-hsd*RMS-*mcr*BC) Φ80*lac*ZΔM15 Δ*lac*X74 *rec*A1 *ara*D139 Δ(*araleu*)7697 *gal*U *gal*K *rps*L (StrR) *end*A1 *nup*G | Invitrogen |
| CC118λpir | Δ(*ara-leu*), *araD*, Δ*lac*X74, *galE*, *galK*, *phoA*, *thi1*, *rpsE*, *rpoB*, *argE* (Am), *recA1*, lysogenic (*λpir*) | (5) |
| *Plasmids* | | |
| pGM931 | pHERD20T derivative; araC/$P_{BAD}$ - $t_\Omega$, $Ap^r$ | (6) |
| pGM-*gssA* | pGM931 derivative; *gssA* under $P_{BAD}$, $Ap^r$ | this work |
| pGM-$gssA_{GUGmut}$ | pGM931 derivative; *gssA mutated CAC→GUG in position 239-241*, under $P_{BAD}$, $Ap^r$ | this work |
| pXG10-SF | sfGFP reporter plasmid; *lacZ*::*gfp* under $P_{LtetO-1}$, $Cm^r$ | (7) |
| pXG10-*hfq::sfGFP* | pXG10-SF derivative; $P_{LtetO-1}$-->*hfq::sfGFP*, $Cm^r$ | this work |
| pBBR1-MCS5 | REP, *lacZ*α, $Gm^r$ | (8) |
| pBBR1-gfp | sfGFP reporter plasmid $P_{LtetO-1}$ -->*sfGFP*, $Gm^r$ | (9) |
| pBBR1-*hfq*::*sfGFP* | pBBR1-MCS5 and pXG10-*hfq*::sf*GFP* derivative; $P_{LtetO-1}$--> *hfq::sfGFP*, $Gm^r$ | this work |
| pSEVA612S | OriR6K, *lacZ*α, MCS flanked by two I-*Sce*I, $Gm^r$ | (10) |
| pSW-1 | OriRK2, *xylS*, *Pm*-->I-*Sce*I, $Ap^r$ | (11) |
| pSEVAPA14-Δ*gssA* | pSEVA612S derivative, $Gm^r$ | this work |
| pSEVAPA14-Δ*hfq* | pSEVA612S derivative, $Gm^r$ | (2) |

## References


1. Rahme LG, Stevens EJ, Wolfort SF, Shao J, Tompkins RG, Ausubel FM. 1995. Common virulence factors for bacterial pathogenicity in plants and animals. Science 268:1899-902.
2. Carloni S, Macchi R, Sattin S, Ferrara S, Bertoni G. 2017. The small RNA ReaL: a novel regulatory element embedded in the *Pseudomonas aeruginosa* quorum sensing networks. Environmental Microbiology 19:4220-4237.
3. Schulz S, Eckweiler D, Bielecka A, Nicolai T, Franke R, Dotsch A, Hornischer K, Bruchmann S, Duvel J, Haussler S. 2015. Elucidation of sigma factor-associated networks in *Pseudomonas aeruginosa* reveals a modular architecture with limited and function-specific crosstalk. PLoS Pathog 11:e1004744.
4. Ferrara S, Carloni S, Fulco R, Falcone M, Macchi R, Bertoni G. 2015. Post-transcriptional regulation of the virulence-associated enzyme AlgC by the σ22-dependent small RNA ErsA of *Pseudomonas aeruginosa*. Environmental Microbiology 17:199-214.
5. de Lorenzo V, Timmis KN. 1994. Analysis and construction of stable phenotypes in gram-negative bacteria with Tn5- and Tn10-derived minitransposons. Methods Enzymol 235:386-405.
6. Delvillani F, Sciandrone B, Peano C, Petiti L, Berens C, Georgi C, Ferrara S, Bertoni G, Pasini ME, Deho G, Briani F. 2014. Tet-Trap, a genetic approach to the identification of bacterial RNA thermometers: application to *Pseudomonas aeruginosa*. RNA 20:1963-76.
7. Corcoran CP, Podkaminski D, Papenfort K, Urban JH, Hinton JC, Vogel J. 2012. Superfolder GFP reporters validate diverse new mRNA targets of the classic porin regulator, MicF RNA. Mol Microbiol 84:428-45.
8. Kovach ME, Elzer PH, Hill DS, Robertson GT, Farris MA, Roop RM, 2nd, Peterson KM. 1995. Four new derivatives of the broad-host-range cloning vector pBBR1MCS, carrying different antibiotic-resistance cassettes. Gene 166:175-6.
9. Ferrara S, Carloni S, Fulco R, Falcone M, Macchi R, Bertoni G. 2015. Post-transcriptional regulation of the virulence-associated enzyme AlgC by the sigma(22) -dependent small RNA ErsA of *Pseudomonas aeruginosa*. Environ Microbiol 17:199-214.
10. Martinez-Garcia E, de Lorenzo V. 2011. Engineering multiple genomic deletions in Gram-negative bacteria: analysis of the multi-resistant antibiotic profile of *Pseudomonas putida* KT2440. Environ Microbiol 13:2702-16.
11. Wong SM, Mekalanos JJ. 2000. Genetic footprinting with mariner-based transposition in *Pseudomonas aeruginosa*. Proc Natl Acad Sci U S A 97:10191-6.
